# The full spectrum of ethical issues in dementia research: findings of a systematic qualitative review

**DOI:** 10.1186/s12910-020-00572-5

**Published:** 2021-03-26

**Authors:** Tim G. Götzelmann, Daniel Strech, Hannes Kahrass

**Affiliations:** 1 Institute for History, Ethics and Philosophy in Medicine, OE 5450, Hannover Medical School, Carl-Neuberg-Str. 1, 30625 Hannover, Germany; 2 QUEST Center, Berlin Institute of Health, Anna-Louisa-Karsch-Straße 2, 10178 Berlin, Germany

**Keywords:** Medical ethics, Research ethics, Ethics, Dementia research, Dementia

## Abstract

**Background:**

When including participants with dementia in research, various ethical issues arise. At present, there are only a few existing dementia-specific research guidelines (Committee for Medicinal Products for Human Use in Clinical investigation of medicines for the treatment Alzheimer’s disease (Internet). https://www.ema.europa.eu/en/clinical-investigation-medicines-treatment-alzheimers-disease; Food and Drug Administration, Early Alzheimer’s Disease: Developing Drugs for Treatment Guidance for Industry [Internet]. http://www.fda.gov/regulatory-information/search-fda-guidance-documents/alzheimers-disease-developing-drugs-treatment-guidance-industy), necessitating a more systematic and comprehensive approach to this topic to help researchers and stakeholders address dementia-specific ethical issues in research. A systematic literature review provides information on the ethical issues in dementia-related research and might therefore serve as a basis to improve the ethical conduct of this research. This systematic review aims to provide a broad and unbiased overview of ethical issues in dementia research by reviewing, analysing, and coding the latest literature on the topic.

**Methods:**

We conducted a systematic review in PubMed and Google Scholar (publications in English between 2007 and 2020, no restrictions on the type of publication) of literature on research ethics in dementia research. Ethical issues in research were identified by qualitative text analysis and normative analysis.

**Results:**

The literature review retrieved 110 references that together mentioned 105 ethical issues in dementia research. This set of ethical issues was structured into a matrix based on the eight major principles from a pre-existing framework on biomedical ethics (Emanuel et al. An Ethical Framework for Biomedical Research. in The Oxford textbook of clinical research ethics, Oxford University Press, Oxford, 2008). Consequently, subcategories were created and further categorized into dementia stages and study phases.

**Conclusions:**

The systematically derived matrix helps raise awareness and understanding of the complex topic of ethical issues in dementia research. The matrix can be used as a basis for researchers, policy makers and other stakeholders when planning, conducting and monitoring research, making decisions on the legal background of the topic, and creating research practice guidelines.

## Background

Dementia prevalence rates are estimated to quadruple by 2050 [1, 2]. Though such forecasts must be interpreted carefully, the global community is likely to face several challenges concerning the individual and familial burdens, societal and political consequences, and economic impact of dementia. With the growing size of the population with dementia, the costs of care are expected to increase in the near future [1].

The need for research on risk factors [2], palliative care, and reducing individual psychological burden is therefore of global importance. Research conducted with participants living with dementia raises important ethical questions, such as how to protect cognitively impaired persons against exploitation, how to design informed consent (IC) procedures with proxies, how to disclose risk-factors for dementia given the lack of evidence for their reliability, and how to apply risk–benefit considerations in such cases [3].

Out of fear of not being able to fulfil the ethical obligations required when conducting research with incapacitated persons, some might suggest the overall exclusion of cognitively impaired persons, or even of all individuals affected by dementia, from research. This caution may lead to the abandonment of meaningful research on dementia and would exclude dementia research from medical progress, leaving affected persons and their relatives orphaned.

Several guidelines [4, 5] provide some orientation as to what should be considered to ensure that research on humans is ethical. These guidelines cover the entire research process from planning, conducting, and monitoring the trial to post-trial. Furthermore, they claim specific protection for vulnerable groups and individuals but are not meant to provide details on what that means for dementia research or other patient groups. Many authors have discussed the ethical challenges of dementia research [3, 6–8, 12, 14–16]. These publications are characterized by a rather narrow focus on certain issues, e.g., on alternatives for obtaining IC [3, 6–13] or genetic testing [14–20]. Some even use a combination of a systematic and a narrative review approach, with the emphasis on identifying differences in the ways ethical issues are addressed [21]; however, a review of the full spectrum of ethical issues in dementia research is still missing in the current literature.

In our systematic review, we therefore aimed to identify the full and unbiased spectrum of research on ethical issues in dementia as discussed in the literature.

## Methods

### Literature search and selection

Three strategies were applied to the literature search: PubMed (database), Google scholar and hand searching methods. We included a publication only if it: (a) described a disease research-specific ethical issue (DREI) in dementia research, (b) did not only relate to ethics in dementia care, and (c) the publication was a peer reviewed journal article or a scientific book (monograph, textbook, edited volume). Methodological quality was no eligibility criteria because of the descriptive approach of our study.

**Fig. 1:**
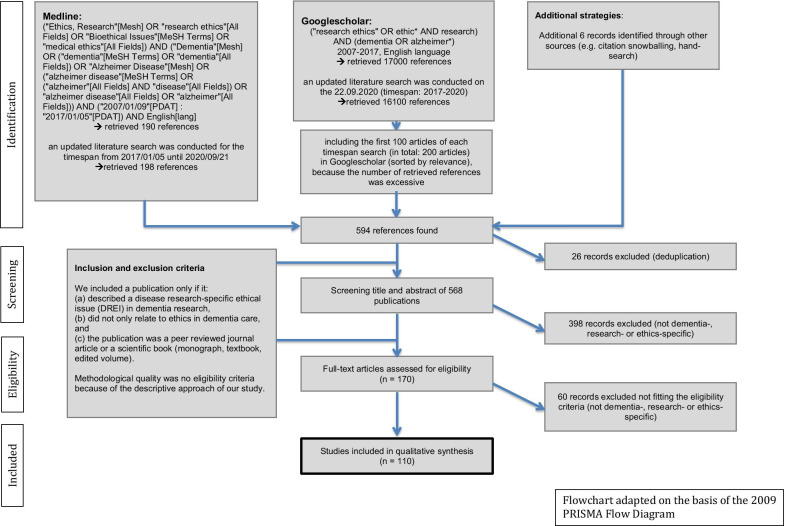
Literature search algorithm adapted on the basis of the 2009 “PRISMA Flow Diagram”

The Flowchart (Fig. 1) presents further details on the search algorithm and the eligibility criteria. This approach has already been applied before and can be read in detail elsewhere [22]. For reference management, we used the programme “Zotero”.

### Definition and typology of dementia research-specific ethical issues (DREIs)

For the definition of DREI, we referred to the ethical theory of principlism. Emanuel et al. suggest eight principles that make clinical research ethical: respect for participants, independent review, fair participant selection/recruiting, favourable risk–benefit ratio, social value, scientific validity, collaborative partnership and IC [4]. These principles represent guiding norms that must be followed in a particular case unless there is a conflict with another obligation that is of equal or greater weight, e.g., alternatives to obtaining IC in special groups or situations. These principles provide only general ethical orientations that require further detail to give guidance in concrete cases. Thus, when applied, the principles must be specified and—if they conflict—balanced against one another.

There are two types of ethical issues that could arise: (a) inadequate consideration of one or more principles (e.g., “risk of insufficiently informing IRBs [institutional review boards] about adequate steps taken to fulfil the ethical obligations of dementia research”) or (b) conflicts between two or more principles (e.g., “challenge of balancing divergent statements in ARD [advance research directive] against current dementia patient wishes or proxy decisions (now vs. then)”). The terms "risk" (a) and "challenge" (b) used in the following refer to this conceptual consideration.

### Analysis and synthesis of DREIs

For analysis, we used thematic content analysis [23] for all 110 included references. To identify and clarify potential ambiguities during content analysis as early as possible a first purposively sampled cluster of references (n = 10) was coded by two reviewers (TG, HK) independently. Another sample of detailed references (n = 9) was coded by one reviewer (TG) only. To capture as many ethical issues as possible this first cluster purposively included more detailed and comprehensive publications. The identified issues were then compared and grouped into the eight principles framework [4] in a consensus process using a programme for qualitative data analysis (“MAXQDA”). Because the consensus process revealed sufficient clarity for how to deal with ambiguous codings the remaining references (n = 45) were randomly split in half and analysed by one author (HK or TG) only. We updated the search in September 2020 and included another sample of 46 studies. These studies were coded by one author (TG). If further ambiguities during coding occurred they were discussed and clarified in the team.

For synthesis, we used a mixed deductive-inductive approach that takes into account the eight principles and the descriptions from the primary literature. We introduced subcategories if we found it reasonable to do so (for example if the number of DREIs was high). Finally, we used *dementia stage* and *study phase* to further categorize the identified issues (see Table 1). While we started with the established eight principles for clinical research ethics as a coding framework, our coding procedure was open for DREIs which could not be grouped under one of the eight principles.

**Table 1:** Principles and issues

*Respect for participants*
Risk that legal protections fail to protect the dementia-related population because existing laws and policies do not apply to non-genetic test results from, e.g., amyloid biomarkers^IV, D^
Risk that there is a lack of guidance for professionals in risk-information disclosure, leading to harm^IV, D^
Risk that participants’ statements signify not a deliberate cognitive act but rather a means of engagement^V, D^
Challenge of imbalance between respecting participant autonomy and protection of the participant^V, D^
Risk of dependency of the participants on relationship with the researcher, which makes an after-trial transition plan/support necessary^V, C^
Risk of harming dementia patient by disclosure of research results (e.g. reporting leading to harm, family disruptions by disclosure of risk information)^V, C^
Challenge of using adequate language, e.g., explicitly referring to the diagnosis as dementia or not, when communicating with the participant^V, D^
Risk that the risk status is revealed by obvious side effects, if experimental drugs are given only to people at (high) risk^I, B^
Risk that disclosure of risk information leading to harm of the study partner/(pre-)caregiver^I, E^
Risk that unexpected end of dementia trial leading to harm^V, C^
Risk of lack of follow up plans for excluded participants at risk for developing dementia leading to serious harm, e.g. suicide^I, C^
*Independent review*
Risk that research ethics committees’ (RECs) and/or IRB’s quality control of consent procedures are not uniform and comparable, e.g. in European countries and the U.S.^V, A^
Risk of RECs weighing opinions of physicians (protecting the participant) over patients’ willingness to participate and over nurse counsellors’ opinions^V, A^
Risk that RECs systematically exclude patients with dementia because of different reservations, e.g., risks are too great; no other options than normal informed consent accepted^IV, A^
*Fair participant selection/recruiting*
Risk of excluding relevant subgroups, e.g. inhabitants of nursing homes, those lacking a proxy/spouse or patients with other psychiatric diseases, from dementia research^IV, A^
Risk of excluding patients with dementia from research due to lack of capacity to consent^IV, A^
Risk that informed consent (IC) is not valid using transparent enrollment (risk marker status-dependent inclusion) because its assessment is mandatory^V, A^
Risk that gatekeepers in dementia research process hinder possible participants in participating in dementia research^IV, A^
Risk that differing national legal frameworks on research leading to exclusion of people with dementia^III, A^
*Favorable risk–benefit ratio*
*Determining risk adequately*
Risk of treating dementia patients unequally because there is no consensus on the definition of ‘minimal risk’^IV, E^
Risk of misconception of risk marker predictive value^IV, D^
Risk of harm due to not-yet-known negative long term effects of disclosing the risk marker status^IV, E^
Risk that risk marker-positive but asymptomatic people will take/receive off-label treatments, e.g. statins, or interventions in the hope of reducing their risk^I, C^
Risk of imbalance in risk–benefit ratio in adaptable trial designs leading to more benefits to later-accessing participants, which could lead to gaming the study by entering later^IV, D^
*Considering risk adequately*
Risk of over diagnosis in asymptomatic persons, if the diagnosis is derived from the risk marker status, since their corresponding validity regarding the occurrence and course of a disease is (still) limited^I, E^
Risk of neglecting the psychological distress of asymptomatic persons caused by disclosure of the risk status^IV, D^
*Managing risks adequately*
Risk of a lack of procedures to minimize harms of risk information disclosure^IV, D^
Risk of discrimination and/or stigmatization against relatives of participants in genetic risk research^I, D^
Risk of possible discrimination of risk marker positive participants^IV, D^
Risk of inadvertent disclosure leading to harm, e.g. in blinded RCTs [randomized controlled trials], in prospective cohort studies^IV, B^
Risk of non-disclosure of information to participants being unduly paternalistic^IV, D^
Risk of participant stigmatization by the use of diagnostic labels such as dementia and mild cognitive impairment (MCI)^IV, E^
Risk of stigmatization by not using value-neutral and label-free language that is less likely to connote abnormality or foster a sense of “otherness.”^IV, D^
Risk of participant discrimination through insurers and employers gathering information about the risk of developing dementia^IV, D^
Risk of risk marker status disclosure leading to misinterpretation of cognitive status and therefore harm^I, B^
*Social value*
Challenge of dealing with the uncertainty of a socially accepted wish to gain knowledge on dementia predisposition^V, E^
Risk of interpreting findings from dementia research incorrectly because of poor reporting, especially details on informed consent^V, C^
*Scientific validity*
*Research design and planning*
Challenge of balancing established standards against personal preferences for certain methodological considerations, e.g., limited number of eligible people in the dementia-related population, when a blinded enrollment is preferred^V, E^
Risk of dementia population fearing possible stigmatization, leading to low participation rates^IV, A^
Risk of poor internal validity because of the heterogeneity of the MCI-population unless this is not compensated by recruiting more participants^V, D^
Risk of compromising external validity in high-risk research by including only participants capable of giving IC^V, D^
*Recruiting bias*
Risk that transparent recruiting and the accompanying diagnostic label causes a smaller and less generalizable pool of potential participants^V, A^
Risk of making high risk research, e.g., a neurosurgical gene transfer trial, impossible in late stage dementia if the consent of a competent person must be in direct chronological connection^V, D^
Risk of recruiting bias when competency to consent is an inclusion criterion leading to a non-representative sample of participants^V, A^
Risk of undue exclusion of participants and jeopardized reproducibility of the study because of ambiguously formulated exclusion criteria that offer researchers too much freedom for selective recruiting^V, A^
Risk that the requirement of a study partner leads to low participation rates^IV, A^
Risk to delay scientific progress when studies fail to recruit adequate numbers of representative participants for AD studies^V, A^
Risk of generating a non representative sample by not including participants which lack a proxy^IV, A^
*Informant bias*
Challenge of including underrepresented subgroups, especially persons living alone, without causing information bias, because here medical history is based on the statements of a person with dementia^IV, A^
Risk of risk information disclosure (e.g., at-risk status) leading to biased cognitive test results of the dementia-related participant (e.g. worsened test result because of negative self assessment)^IV, D^
Risk of getting inadequate information about the medical history important for research from a person diagnosed with dementia because of the cognitive decline^IV, D^
Risk that data provided by proxies differ from actual participants’ opinion^IV, D^
*Drop-outs*
Risk that dementia patients experiencing stigmatization will lead to low follow-up rates or study withdrawal^IV, B^
Risk that dementia prevention studies without participant study partners leading to higher dropouts leading to lower statistical power^IV, B^
Risk that non-spousal research dyads lead to lower completion rates in AD studies^IV, B^
*Agenda setting*
Risk of imbalanced research, because today, studies on dementia types with small prevalence are conducted more often than studies of other types^V, E^
Risk of hindering international dementia research because implementations of EU[European union]-guidelines differ on a national level^V, A^
*Collaborative partnership*
Risk of caregiver misrepresenting participants’ statements when they are consulted because of their knowledge of the participant^IV, D^
Risk of lack of communication between researcher and possible dementia study population leading to selection^IV, D^
Risk of reduced value of dementia research if different perspectives on dementia research are not taken into account^V, E^
Risk of hindering public debate on dementia research because of a non-uniform language/communication^V, E^
Risk that professional guidelines are not based on a broad empirical background, especially concerning lay people dealing with dementia^V, E^
*Informed consent*
*Qualified personnel*
Risk that less-experienced researchers will lack the skills necessary for a sensible and adequate handling of the challenges that appear in the informed consent process in research with dementia patients^IV, A^
*Good guidance*
Risk of overgeneralization of specific problems and low focus on dementia patients because of a lack of dementia-specific guidelines on IC issues^IV, D^
*Right (amount of) information*
Risk of uncertainty about what to disclose to the participant because there is no clear guidance on what risk information should be disclosed to the dementia patient^IV, D^
Risk of undermined IC because of lack of information on efficacy and safety in deep brain stimulation studies^IV, A^
Challenge of balancing the intention not to harm participants by using stigmatizing diagnostic labels (such as dementia or MCI) and IC being not valid in such cases because of lack of information^IV, A^
*Understanding*
Risk of therapeutic misconception of pre-symptomatic dementia patients (e.g., biomarker positive or pet-ct [Positron emission tomography–computed tomography] positive populations)^IV, D^
Risk of therapeutic misconception of dementia patients applying for deep brain stimulation studies^IV, A^
Risk of therapeutic misconception being higher in participants with MCI or mild dementia^IV, A^
*Capacity assessment*
Risk that capabilities of participants will be overestimated, especially if the patients are not yet completely incompetent^IV, D^
Risk that cognitive assessment tests (e.g. MMSE [mini mental state examination]) in dementia research are harmful because they focus on people’s deficits rather than their strengths^IV, D^
Risk of confusing the expressed willingness of a dementia patient to participate in research with the capacity to consent^III, A^
Risk of taking a diagnosis of dementia as an exclusion criterion without considering the actual competency of the patient^IV, A^
Risk that cognitive assessment tests in dementia research lack a final determination of capacity^IV, A^
*Obtaining informed consent (incl. safeguards)*
Risk that the obligation of proxy consent in dementia research slows down the recruitment process and can endanger scientific validity^III, A^
Risk of misjudging the actual meaning of a patients expression of dissent regarding study participation^IV, B^
Risk of excluding dementia patients because of a too-rigid study design that could not wait for a "good day" to include a person^V, A^
Risk of IC process in first in human studies is invalid because of vulnerability of research participants^IV, A^
Risk of IC being insufficient to safeguard confidentiality in regard to big data approaches in dementia research^IV, D^
Risk of conflict of interest if researcher has the decision-making authority over the participant^IV, D^
*Proxy consent*
Risk that in dementia research, proxy feels unable to decide if no written advanced research directive (ARD) exists^III, A^
Challenge of balancing divergent statements in ARD against current dementia patient wishes or proxy decisions (now vs. then)^IV, D^
Risk of focusing only on proxy in consent process and neglecting the person with dementia^III, D^
Risk that proxy consent in dementia research is limited to therapeutic research and non-therapeutic research with minimal risk and minimal burden^III, D^
Risk that varying (inter)national regulations are a burden for (inter)national dementia research^IV, E^
Risk that proxy consent in dementia research becomes more difficult to achieve with increasing numbers of possible proxies in a family^III, D^
Risk of not considering that proxies have major self interest in dementia research, e.g., because they have same genetic traits, which could influence their proxy decision, and their manipulative behavior may be difficult to detect^III, D^
Risk that proxy of a dementia patient misunderstands aspects of research, e.g., therapeutic misconception^III, D^
Risk of proxy consent in dementia research being a moral burden for legal representative^III, D^
Risk that proxy consent in genetic dementia research in larger families might violate the right-not-to-know of individuals if every family member is not included in the consent process^IV, D^
Risk of not fulfilling the obligation of IC by only obtaining proxy consent in first-in-human studies^IV, D^
*Broad consent*
Risk that broad consent leading to harm, e.g. privacy issues concerning inadequate data use^V, D^
*Advance research directives (ARDs)*
Risk of confusion concerning ARD issues because of no existing guidelines towards ARDs in dementia research^IV, D^
Risk that ARD in dementia research cannot be a truly informed decision because of the impossibility of anticipating a situation never experienced^IV, A^
Risk that new information (“body of evidence”) presents an obstacle to the interpretation of ARDs in dementia research^III, D^
Risk that ARDs have no practical relevance—popular in theory but not used in high numbers in dementia research^IV, E^
*Ongoing assessment*
Risk that IC at the beginning of a dementia study alone is insufficient because of cognitive decline of participants^IV, A^
Challenge of dealing with varying standards/thresholds to determine and re-evaluate competency/capacity^IV, D^
Risk of not monitoring signs of distress throughout data collection to satisfy the core idea of IC, especially if no standard IC procedure was possible because of the participant’s dementia^IV, B^
Risk of not being able to differentiate dissent from symptoms of dementia^IV, D^
Risk that possible ‘direct benefit considerations’ will lead to overstepping the ‘right to dissent at any time’ in a dementia study^IV, B^
*Informed consent document*
Risk that simplified IC forms will lead to psychological distress for dementia patients^IV, D^
*Ethical oversight*
Risk of insufficiently informing IRBs about adequate steps taken to fulfill the ethical obligations of dementia research^V, A^
Risk that uncertainties about consent in dementia research will lead to problems in the participation in longitudinal studies^IV, D^

Dementia stage: ^I^ = cognitively unimpaired; ^II^ = mild cognitive impairment; ^III^ = dementia; ^IV^ = overarching I, II, III; ^V^ = unspecified

Study phase: ^A^ = Recruiting/pre-trial; ^B^ = conduction phase; ^C^ = post-trial; ^D^ = general; ^E^ = unspecified

## Results

### References and journals

The literature search in PubMed and Google Scholar revealed a total set of 594 references, 110 of which were ultimately included in the analysis, published between 2007 and 2020 in 64 different journals. For more details, see the flowchart (Fig. 1).

### Spectrum of dementia research ethical issues (DREIs)

The analysis of the 110 references revealed 105 DREIs. All identified issues could be grouped under one of the eight principles for ethical research, some having far more DREIs than others. In detail, “respect for participants” (n = 11 DREIs), “independent review” (n = 3), “fair participant selection/recruiting” (n = 5), “favourable risk–benefit ratio” (n = 16, 3 subcategories), “social value” (n = 2), “scientific validity” (n = 20, 5 subcategories), “collaborative partnership” (n = 5) and “informed consent (IC)” (n = 43, 12 subcategories). In the course of data analysis, we subsequently found fewer new codes, and the last 10 analysed papers raised no new issues. Thus, we appear to have achieved thematic saturation for the spectrum at least for the level of major groups and first-level subgroups. We updated the search in September 2020 which lead to the analysis of 46 references from the years 2017 until 2020. During the process of literature analysis, only one new subcategory was found in a paper from 2018 [24], hereafter no new sub-categories have been identified (for the years of 2019 and 2020).

All identified DREIs and subcategories are presented in Table 1. This table also contains the categorization according to the *dementia stage* (based on the NIA-AA-2018-Framework) [25] and the *phase of the research* for each issue, symbolized by superscript numbers or characters. Additionally, the 105 DREIs are presented in separate tables for each category of *dementia stage* (Additional file 1) and the *phase of the research* (Additional file 2). A full list of the found issues together with the accompanying original text examples as well as the list of all references that were analysed during our systematic review are available in Additional file 3: Table S3. The above listed tables are available at the supplemental data.

We used the nomenclature of the NIA-AA-2018-framework (“cognitively impaired”, “mild cognitive impairment (MCI)” and “dementia”) [25] for the first three categories in our *dementia stages* categorization. Most DREIs were related to more than one dementia stage (category IV, n = 60, Table 1). DREIs related to “cognitively unimpaired” (category I, n = 7) centre around the principle of favourable risk–benefit ratio, especially dealing with the sub-categories “determining risk adequately” and “considering risk adequately”, and the principle of respect for participants. No issues were found to fit “mild cognitive impairment” exclusively (category II), where people with dementia are not yet incapacitated. In category III = dementia (n = 11), issues mostly referred to “IC”, especially addressing the sub-category “proxy consent”. Finally, 27 DREIs could not be classified in that split spectrum.

Concerning the categorization due to *study phase*, we used a timeline approach in naming the different study phases (I = recruiting/pre-trial, II = conduction phase, III = post-trial, IV = general). For DREIs related to specific study phases, again, most DREIs were found to be of overarching relevance (category D, n = 45). In the recruiting/pre-trial phase, DREIs arise within “independent review”, “fair participant selection/recruiting”, “scientific validity”, and “informed consent” (category A, n = 32). While conducting the study (category B, n = 9), DREIs are related to “drop-outs” that endanger scientific validity and the “ongoing assessment” within the principle of informed consent. The post-trial phase is mostly concerned with the principle of respect for participants, communicating the results to the participants and the scientific community (“poor reporting quality”) and adequate follow-up of the volunteers (category C, n = 6). Thirteen issues could not be classified under the topic of *study phase*.

### Specification of general principles for ethical dementia research

All principles for ethical research [4] were specified in the analysed literature. The references to general principles, such as “IC”, are rather implicit; however, authors elaborate on how the characteristics of dementia lead to specific ethical challenges, e.g., “However, a special ethical issue with regard to longitudinal studies that end in participants’ death is that participants are competent when first recruited, but have a significant likelihood of becoming incompetent while they are study subjects. […] [T]he gradual loss of the capacity to consent […] creates challenges for informed consent, the ethical bedrock of research with human subjects. […][Here], it may make sense to re-evaluate consent capacity […] at several intervals during the study" [6].

From this statement, the following DREI was paraphrased: “Risk that IC at the beginning of a dementia study alone is insufficient because of cognitive decline of participants”. This DREI is of general relevance for all dementia stages but has particular relevance to the study phase “recruiting/pre-trial”. The full spectrum of issues, including original text examples and all references, is presented in the online supplement (see Additional file 3).

Issues which were mentioned the most, are, for example, “Risk of excluding relevant subgroups, e.g. inhabitants of nursing homes, those lacking a proxy/spouse or patients with other psychiatric diseases, from dementia research” (n = 25 papers) and “Risk of excluding participants from research due to lack of capacity to consent” (n = 23). Examples of rarely mentioned DREI are “Risk that dementia patients experiencing stigmatization will lead to low follow-up rates or study withdrawal” (n = 1), “Risk of therapeutic misconception being higher in participants with MCI or mild dementia” (n = 1) and “Risk of RECs [research ethics committees] weighing opinions of physicians (protecting the participant) over patients’ willingness to participate and over nurse counsellors’ opinions” (n = 1).

Several DREIs were only addressed in an implicit manner; for example, “Risk that varying international regulations are a burden for international dementia research” is based on the following quotation: “However, only in Germany and Italy is the system of proxy determined by the courts—a procedure which is not necessarily required for the recognition of a proxy in other member states” [26].

## Discussion

This systematic literature review identified and synthesized the full spectrum of 105 ethical issues in dementia research (DREIs) based on 110 references published between 2007 and 2020 in 64 different journals.

Many ethical issues involved “IC” (n = 11) in incapacitated participants and “risk-information disclosure” (n = 8). However, this review shows that there are many more DREIs to consider when planning, reviewing, conducting, or monitoring research with this vulnerable group. We assume that the results will be of interest to different groups—clinical experts, researchers, policy makers, REC-members, lawyers, patient-organization representatives or even affected persons themselves—and that the different stakeholders will read and use the results differently.

Our review lists several ethical issues grouped under eight broadly established ethical principles for clinical research. These principles and the principlism approach in general are correlative to basic human rights [26]. The eight principles are not focused on capacity-based approaches but include approaches to express the right to participate in research via, for examples, advance directives. We would therefore argue, in line with many other ethical analyses based on a principlism approach, that human rights related ethical issues in dementia research are captured directly and indirectly by the many ethical issues addressed in our list of issues. The same applies to other overarching normative concepts such as “avoiding exploitation”. No specified ethical issue in our list mentions the risk of exploitation directly but more or less all specific ethical issues address this risk indirectly. Likewise, the wording “human rights” did not appear explicitly in the literature we analyzed.

Those looking for support or guidance on how to seek ethically appropriate dementia research might prefer detailed descriptions of very specific challenges. Articles such as “Seeking Assent and Respecting Dissent in Dementia Research” by Black et al. [9] serve this purpose. However, these publications often focus on particular aspects and do not aim to provide a detailed and systematic overview. Further, one also has to do thorough searching and read a large volume of material (we screened n = 594 and finally included n = 110 references) to be familiar with all the aspects discussed in the literature. In contrast to literature addressing very specific DREIs, there are also broad, theoretical frameworks for research ethics, such as that of Emanuel et al. [4]. However, if capacity building for ethics in dementia research is primarily informed by such general frameworks, it might overlook issues that only become apparent when specifying practice-related tasks. Our review is intended to bridge detailed specifications with a comprehensive and structured presentation of the DREIs at stake.

We illustrate the bridging character of our study by comparing one benchmark for the IC principle originating from Emanuel et al.’s framework [4] with one issue on our spectrum grouped under “IC” in the subcategory “proxy consent”. The benchmark is “Are there appropriate plans in place for obtaining permission from legally authorized representatives for individuals unable to consent for themselves?” [4]. A researcher with a specific trial in mind would, in order to conduct morally sound research, perhaps refer to that benchmark in a case where they plans to start a trial on incapacitated patients suffering from dementia. This person would then fulfil that benchmark by making it possible for legal representatives of the patients to fill out the IC document in place of the incapacitated participant. Thus, they would fulfil the benchmark and might not think about more specific ethical problems that might arise when one looks into the literature describing DREI. One such example is this quote stemming from an article on dementia research ethics: “Proxy consent, already an issue of debate in traditional research, was considered more problematic in genetic research, where children share the same genetic traits as their parents. On the one hand, this might be a motivation for the affected parent to participate in a research study to help their children. On the other hand, it was questioned that to what extent children still are able to make a decision in the best interest of their parents because they have an interest themselves. The more genetic research will be carried out, the higher the chance on a disease modifying or preventive therapy for them and their children” [14].

In that case, and if the researcher had a plan to conduct research in this field of genetic dementia research, the simple fulfilment of the abovementioned benchmark would be insufficient for the goal of morally acceptable research. The mentioned quotation informed the creation of the DREI “Risk of not considering that proxies have major self interest in dementia research, e.g., because they have same genetic traits, which could influence their proxy decision, and their manipulative behaviour may be difficult to detect”.

As we compared topics between both rounds of the literature analysing process, we noticed, that some topics were newly introduced in the scientific literature, in particular “deep brain stimulation” issues in dementia research. Other categories or sub-categories like “social value”, “qualified personnel” and “informed consent document” were not further discussed in scientific literature.

In addition, we found more and more text examples for issues which before the year of 2017 were only mentioned once, e.g. “Risk of over diagnosis in asymptomatic persons, if the diagnosis is derived from the risk marker status, since their corresponding validity regarding the occurrence and course of a disease is (still) limited”, which now was mentioned in six papers.

Also, in the course of the analysis of the studies between 2017 and 2020 we found 22 new issues, among them 17 issues which were only mentioned by one paper showing the rapid emergence of new issues in the dementia research ethics field.

Capturing this full spectrum of DREIs can serve multiple purposes. First, it can raise awareness of the ethical issues arising in the context of dementia research, highlighting issues that may be underrepresented in the published literature through the side-by-side presentation in our matrix. Second, it can serve as the basis for information or training materials for researchers and caregivers. Third, it can form the basis for discussions on the importance and/or relevance of the different ethical issues. Fourth, because our spectrum does not rank the difficult DREIs in order of importance, third parties can use it as a basis for exactly that purpose. Fifth, developers of specific research guidelines or policy papers may use this spectrum as an entry point to that topic.

At this point, it is important to state that our spectrum remains strictly descriptive. The qualitative and normative interpretation is therefore left to others, e.g., researchers, policy-makers, patient organizations, funding partners and the community as a whole. Those interpretations could further help in developing stakeholder-oriented guidelines for conducting ethically sound research in dementia. The list of ethical issues as presented in this paper, however, cannot directly serve as a checklist for review purposes. More conceptual work is needed to translate the in-depth results of this systematic review into effective and efficient normative or procedural guidance. Finally, existing guidelines, policy papers or new research articles on the topic of DREIs can be screened for completeness [27].

To make the results of the review more concise and accessible, we prepared overviews sorted by *stage* and *phase* (available as an online supplement). This is particularly suitable for readers who have a certain focus, e.g., because they are currently planning a study with people in an early stage of dementia (see Additional file 1) or are looking for an overview of DREIs in the phase of conducting the study (see Additional file 2). These tables show that ethical issues are situation-sensitive, e.g., certain questions on informed consent only arise at a later stage of the disease, while questions of reporting the status of risk factors are only relevant in early stage (pre-symptomatic) patients.

One limitation of this systematic review is that the search was limited to PubMed and Google Scholar. We do not consider this an overly disadvantageous factor and consider the approach to be appropriate for the following reasons: First, our search resulted in the identification of literature from different fields, not only from the bioethics and medicine field but also spanning nursing research [28, 29], nursing ethics [30, 31], a narrative review [3] and even one systematic review [21]. This systematic review by West et al. covered mostly literature concerning IC, advance directives and the role of proxies or surrogates. Second, thematic saturation for the first-level categories was achieved after analysing 54 of the 64 papers that were included after the first literature search in these two data sources. For the updated literature search, which only found one new first-level category, thematic saturation was achieved after analysing 25 of the 46 papers. Third, former systematic reviews [32, 33] in the bioethics field, which based their research on additional databanks such as EMBASE, CINAHL or Euroethics, found few additional references. Another limitation is that we only reviewed the literature from the last 14 years. However, we included two (systematic) reviews, which included literature dating back to 1982 [3] and back to 1995 [21]. We further assume that an important ethical issue that was mentioned 15 years ago and that is still relevant nowadays would be addressed in some more recent references again.

Further, we only included references in the English language. Some culturally sensitive DREI might be preferably discussed in the respective language, and our review might have missed those discussions. Last but not least, we only included peer-reviewed literature and thus did not consider grey literature such as guidelines from advocacy organizations involved with dementia research [34, 35]. As a future project, we aim to employ the results of our review to analyse whether and how guidelines for dementia research mention the identified issues. For a similar approach see the results of a systematic review of ethical issues in dementia care [22] that was followed-up by a content analysis of clinical practice guidelines for dementia care [27].

The authors of this review have different scientific backgrounds: medicine/psychiatry, physiotherapy, public health, ethics and philosophy. However, all authors are currently involved neither in clinical research nor in health care for people with dementia. However, we do not consider this to be a weakness of the review, as we have included these perspectives in the literature considered, e.g., expert opinions [9, 10, 14, 36–39], views of patients, caregivers and proxies [11, 28, 40–49], papers focusing on legal and ethical guidelines [50–57], and the point of view of lay persons [13]. Our review found no papers on the opinions and views of relatives of people living with dementia. This might indicate the need for further research in that field.

## Conclusions

This study has successfully shown that a systematic literature review leads to a wider spectrum of DREIs (n = 105) than other papers on the subject. The identified issues are specifications of eight general ethical principles for clinical research and could be categorized according to the dementia stage and study phase. Therefore, the spectrum can be used to raise awareness about the complexity of ethics in this field and can support different stakeholders in the implementation of ethically appropriate dementia research.

## Supplementary Information

**Additional file 1. Table S1**: An overview of the 105 DREIs assigned to dementia stages.

**Additional file 2. Table S2**: An overview of the 105 DREIs assigned to study phases.

**Additional file 3. Table S3**: All principles, issues and text examples in one table.

## Data Availability

The datasets used and analysed during the current study are available from the corresponding author on reasonable request.

## Abbreviations

DREI(s): Dementia research-specific ethical issue(s)
IC: Informed consent
IRB(s): Institutional review board(s)
ARD: Advanced research directives
TG: Tim Götzelmann
HK: Hannes Kahrass
MCI: Mild cognitive impairment
REC(s): Research ethics committee(s)
DS: Daniel Strech
RCT(s): Randomized controlled trial(s)
EU: European union
PET-CT: Positron emission tomography-computed tomography
MMSE: Mini-Mental State Examination
