# Supplementary material for: The full spectrum of ethical issues in dementia research: findings of a systematic qualitative review
Source: BMC Med Ethics. 2021 Mar 26;22:32. doi: 10.1186/s12910-020-00572-5 (PMC8004446; doi:10.1186/s12910-020-00572-5)
Supplement: Supplementary file 2 — Additional file 2. Table S2: An overview of the 105 DREIs assigned to study phases. [file 12910_2020_572_MOESM2_ESM.docx]

**Study Phases** (tables S2)

*Sub-table S2.1 – “****recruiting/pre-trial***“

| **Respect for participants** |
| --- |
| None* |
| **Independent review** |
| Risk that research ethics committees’ (RECs) and/or IRB’s quality control of consent procedures are not uniform and comparable, e.g. in European countries and the U.S. |
| Risk of RECs weighing opinions of physicians (protecting the participant) over patients’ willingness to participate and over nurse counsellors’ opinions |
| Risk that RECs systematically exclude patients with dementia because of different reservations, e.g., risks are too great; no other options than normal informed consent accepted |
| **Fair participant selection/recruiting** |
| Risk of excluding relevant subgroups, e.g. inhabitants of nursing homes, those lacking a proxy/spouse or patients with other psychiatric diseases, from dementia research |
| Risk of excluding patients with dementia from research due to lack of capacity to consent |
| Risk that informed consent (IC) is not valid using transparent enrollment (risk marker status-dependent inclusion) because its assessment is mandatory |
| Risk that gatekeepers in dementia research process hinder possible participants in participating in dementia research |
| Risk that differing national legal frameworks on research leading to exclusion of people with dementia |
| **Favorable risk-benefit ratio** |
| None* |
| **Social value** |
| None* |
| **Scientific validity** |
| Research design & planning |
| Risk of dementia population fearing possible stigmatization, leading to low participation rates |
| Recruiting bias |
| Risk that transparent recruiting and the accompanying diagnostic label causes a smaller and less generalizable pool of potential participants |
| Risk of recruiting bias when competency to consent is an inclusion criterion leading to a non-representative sample of participants |
| Risk of undue exclusion of participants and jeopardized reproducibility of the study because of ambiguously formulated exclusion criteria that offer researchers too much freedom for selective recruiting |
| Risk that the requirement of a study partner leads to low participation rates |
| Risk to delay scientific progress when studies fail to recruit adequate numbers of representative participants for AD studies |
| Risk of generating a non representative sample by not including participants which lack a proxy |
| Informant bias |
| Challenge of including underrepresented subgroups, especially persons living alone, without causing information bias, because here medical history is based on the statements of a person with dementia |
| Agenda setting |
| Risk of hindering international dementia research because implementations of EU-guidelines differ on a national level |
| **Collaborative partnership** |
| None* |
| **Informed consent** |
| Qualified personal |
| Risk that less-experienced researchers will lack the skills necessary for a sensible and adequate handling of the challenges that appear in the informed consent process in research with dementia patients |
| Right (amount of) information |
| Risk of undermined IC because of lack of information on efficacy and safety in deep brain stimulation studies |
| Challenge of balancing the intention not to harm participants by using stigmatizing diagnostic labels (such as dementia or MCI) and IC being not valid in such cases because of lack of information |
| Understanding |
| Risk of therapeutic misconception of dementia patients applying for deep brain stimulation studies |
| Risk of therapeutic misconception being higher in participants with MCI or mild dementia |
| Capacity assessment |
| Risk of confusing the expressed willingness of a dementia patient to participate in research with the capacity to consent |
| Risk of taking a diagnosis of dementia as an exclusion criterion without considering the actual competency of the patient |
| Risk that cognitive assessment tests in dementia research lack a final determination of capacity |
| Obtaining IC (incl. safeguards) |
| Risk that the obligation of proxy consent in dementia research slows down the recruitment process and can endanger scientific validity |
| Risk of excluding dementia patients because of a too-rigid study design that could not wait for a "good day" to include a person |
| Risk of IC process in first in human studies is invalid because of vulnerability of research participants |
| Proxy consent |
| Risk that in dementia research, proxy feels unable to decide if no written advanced research directive (ARD) exists |
| Advance research directives (ARD) |
| Risk that ARD in dementia research cannot be a truly informed decision because of the impossibility of anticipating a situation never experienced |
| Ongoing assessment |
| Risk that IC at the beginning of a dementia study alone is insufficient because of cognitive decline of participants |
| Ethical oversight |
| Risk of insufficiently informing IRBs about adequate steps taken to fulfill the ethical obligations of dementia research |
| ** = if you do not find an issue under one principle in one of the sub-tables II.1-3 you might check sub-table II.4 for issues which concern more than one dementia stage* |

*Sub-table S2.2 – “****conduction phase****”*

| **Respect for participants** |
| --- |
| Risk that the risk status is revealed by obvious side effects, if experimental drugs are given only to people at (high) risk. |
| **Independent review** |
| None* |
| **Fair participant selection/recruiting** |
| None* |
| **Favorable risk-benefit ratio** |
| Managing risks adequately |
| Risk of inadvertent disclosure leading to harm, e.g. in blinded RCTs, in prospective cohort studies |
| Risk of risk marker status disclosure leading to misinterpretation of cognitive status and therefore harm |
| **Social value** |
| None* |
| **Scientific validity** |
| Drop-outs |
| Risk that dementia patients experiencing stigmatization will lead to low follow-up rates or study withdrawal |
| Risk that dementia prevention studies without participant study partners leading to higher dropouts leading to lower statistical power |
| Risk that non-spousal research dyads lead to lower completion rates in AD studies |
| **Collaborative partnership** |
| None* |
| **Informed consent** |
| Obtaining IC (incl. safeguards) |
| Risk of misjudging the actual meaning of a patients expression of dissent regarding study participation |
| Ongoing assessment |
| Risk of not monitoring signs of distress throughout data collection to satisfy the core idea of IC, especially if no standard IC procedure was possible because of the participant’s dementia |
| Risk that possible ‘direct benefit considerations’ will lead to overstepping the ‘right to dissent at any time’ in a dementia study |
| ** = if you do not find an issue under one principle in one of the sub-tables II.1-3 you might check sub-table II.4 for issues which concern more than one dementia stage* |

*Sub-table S2.3 – “****post-trial****”*

| **Respect for participants** |
| --- |
| Risk of dependency of the participants on relationship with the researcher, which makes an after-trial transition plan/support necessary |
| Risk of harming dementia patient by disclosure of research results (e.g. reporting leading to harm, family disruptions by disclosure of risk information) |
| Risk that unexpected end of dementia trial leading to harm |
| Risk of lack of follow up plans for excluded participants at risk for developing dementia leading to serious harm, e.g. suicide |
| **Independent review** |
| None* |
| **Fair participant selection/recruiting** |
| None* |
| **Favorable risk-benefit ratio** |
| Determining risk adequately |
| Risk that risk marker-positive but asymptomatic people will take/receive off-label treatments, e.g. statins, or interventions in the hope of reducing their risk |
| **Social value** |
| Risk of interpreting findings from dementia research incorrectly because of poor reporting, especially details on informed consent |
| **Scientific validity** |
| None* |
| **Collaborative partnership** |
| None* |
| **Informed consent** |
| None* |
| ** = if you do not find an issue under one principle in one of the sub-tables II.1-3 you might check sub-table II.4 for issues which concern more than one dementia stage* |

*Sub-table S2.4 – “****general****”*

| **Respect for participants** |
| --- |
| Risk that legal protections fail to protect the dementia-related population because existing laws and policies do not apply to non-genetic test results from, e.g., amyloid biomarkers |
| Risk that there is a lack of guidance for professionals in risk-information disclosure, leading to harm |
| Risk that participants’ statements signify not a deliberate cognitive act but rather a means of engagement |
| Challenge of imbalance between respecting participant autonomy and protection of the participant |
| Challenge of using adequate language, e.g., explicitly referring to the diagnosis as dementia or not, when communicating with the participant |
| Risk that disclosure of risk information leading to harm of the study partner/(pre-)caregiver |
| **Independent review** |
| None* |
| **Fair participant selection/recruiting** |
| None* |
| **Favorable risk-benefit ratio** |
| Determining risk adequately |
| Risk of misconception of risk marker predictive value |
| Risk of imbalance in risk-benefit ratio in adaptable trial designs leading to more benefits to later-accessing participants, which could lead to gaming the study by entering later |
| Considering risk adequately |
| Risk of neglecting the psychological distress of asymptomatic persons caused by disclosure of the risk status |
| Managing risks adequately |
| Risk of a lack of procedures to minimize harms of risk information disclosure |
| Risk of discrimination and/or stigmatization against relatives of participants in genetic risk research |
| Risk of possible discrimination of risk marker positive participants |
| Right (amount of) information |
| Risk of uncertainty about what to disclose to the participant because there is no clear guidance on what risk information should be disclosed to the dementia patient |
| Understanding |
| Risk of therapeutic misconception of pre-symptomatic dementia patients (e.g., biomarker positive or pet-ct positive populations) |
| Capacity assessment |
| Risk that capabilities of participants will be overestimated, especially if the patients are not yet completely incompetent |
| Risk that cognitive assessment tests (e.g. MMSE) in dementia research are harmful because they focus on people’s deficits rather than their strengths |
| Proxy consent |
| Challenge of balancing divergent statements in ARD against current dementia patient wishes or proxy decisions (now vs. then) |
| Risk of focusing only on proxy in consent process and neglecting the person with dementia |
| Risk that proxy consent in dementia research is limited to therapeutic research and non-therapeutic research with minimal risk and minimal burden |
| Risk that proxy consent in dementia research becomes more difficult to achieve with increasing numbers of possible proxies in a family |
| Risk of non-disclosure of information to participants being unduly paternalistic ^IV, D^ |
| Risk of stigmatization by not using value-neutral and label-free language that is less likely to connote abnormality or foster a sense of “otherness.” |
| Risk of participant discrimination through insurers and employers gathering information about the risk of developing dementia |
| Risk of not fulfilling the obligation of IC by only obtaining proxy consent in first-in-human studies |
| Broad consent |
| Risk that broad consent leading to harm, e.g. privacy issues concerning inadequate data use |
| **Social value** |
| None* |
| **Scientific validity** |
| Research design & planning |
| Risk of poor internal validity because of the heterogeneity of the MCI-population unless this is not compensated by recruiting more participants |
| Risk of compromising external validity in high-risk research by including only participants capable of giving IC |
| Recruiting bias |
| Risk of making high risk research, e.g., a neurosurgical gene transfer trial, impossible in late stage dementia if the consent of a competent person must be in direct chronological connection |
| Informant bias |
| Risk of risk information disclosure (e.g., at-risk status) leading to biased cognitive test results of the dementia-related participant (e.g. worsened test result because of negative self assessment) |
| Risk of getting inadequate information about the medical history important for research from a person diagnosed with dementia because of the cognitive decline |
| Risk that data provided by proxies differ from actual participants’ opinion |
| **Collaborative partnership** |
| Risk of caregiver misrepresenting participants’ statements when they are consulted because of their knowledge of the participant |
| Risk of lack of communication between researcher and possible dementia study population leading to selection |
| **Informed consent** |
| Good guidance |
| Risk of overgeneralization of specific problems and low focus on dementia patients because of a lack of dementia-specific guidelines on IC issues |
| Risk of not considering that proxies have major self interest in dementia research, e.g., because they have same genetic traits, which could influence their proxy decision, and their manipulative behavior may be difficult to detect |
| Risk that proxy of a dementia patient misunderstands aspects of research, e.g., therapeutic misconception |
| Risk of proxy consent in dementia research being a moral burden for legal representative |
| Risk that proxy consent in genetic dementia research in larger families might violate the right-not-to-know of individuals if every family member is not included in the consent process |
| Obtaining IC (incl. safeguards) |
| Risk of IC being insufficient to safeguard confidentiality in regard to big data approaches in dementia research |
| Risk of conflict of interest if researcher has the decision-making authority over the participant |
| Advance research directives (ARD) |
| Risk of confusion concerning ARD issues because of no existing guidelines towards ARDs in dementia research |
| Risk that new information (“body of evidence”) presents an obstacle to the interpretation of ARDs in dementia research |
| Ongoing assessment |
| Challenge of dealing with varying standards/thresholds to determine and re-evaluate competency/capacity |
| Risk of not being able to differentiate dissent from symptoms of dementia |
| Informed consent document |
| Risk that simplified IC forms will lead to psychological distress for dementia patients |
| Ethical oversight |
| Risk that uncertainties about consent in dementia research will lead to problems in the participation in longitudinal studies |
| ** = if you do not find an issue under one principle in one of the sub-tables II.1-3 you might check sub-table II.4 for issues which concern more than one dementia stage* |

*Sub-table S2.5 – “****unspecified****”*

| **Respect for participants** |
| --- |
| None* |
| **Independent review** |
| None* |
| **Fair participant selection/recruiting** |
| None* |
| **Favorable risk-benefit ratio** |
| Determining risk adequately |
| Risk of treating dementia patients unequally because there is no consensus on the definition of ‘minimal risk’ |
| Risk of harm due to not-yet-known negative long term effects of disclosing the risk marker status |
| Considering risk adequately |
| Risk of over diagnosis in asymptomatic persons, if the diagnosis is derived from the risk marker status, since their corresponding validity regarding the occurrence and course of a disease is (still) limited |
| Managing risks adequately |
| Risk of participant stigmatization by the use of diagnostic labels such as dementia and mild cognitive impairment (MCI) |
| **Social value** |
| Challenge of dealing with the uncertainty of a socially accepted wish to gain knowledge on dementia predisposition |
| **Scientific validity** |
| Research design & planning |
| Challenge of balancing established standards against personal preferences for certain methodological considerations, e.g., limited number of eligible people in the dementia-related population, when a blinded enrollment is preferred |
| Agenda setting |
| Risk of imbalanced research, because today, studies on dementia types with small prevalence are conducted more often than studies of other types |
| **Collaborative partnership** |
| Risk of reduced value of dementia research if different perspectives on dementia research are not taken into account |
| Risk of hindering public debate on dementia research because of a non-uniform language/communication |
| Risk that professional guidelines are not based on a broad empirical background, especially concerning lay people dealing with dementia |
| **Informed consent** |
| Proxy consent |
| Risk that varying (inter)national regulations are a burden for (inter)national dementia research |
| Advance research directives (ARD) |
| Risk that ARDs have no practical relevance - popular in theory but not used in high numbers in dementia research |
| ** = if you do not find an issue under one principle in one of the sub-tables II.1-3 you might check sub-table II.4 for issues which concern more than one dementia stage* |
