# Supplementary material for: The full spectrum of ethical issues in dementia research: findings of a systematic qualitative review
Source: BMC Med Ethics. 2021 Mar 26;22:32. doi: 10.1186/s12910-020-00572-5 (PMC8004446; doi:10.1186/s12910-020-00572-5)
Supplement: Supplementary file 3 — Additional file 3. Table S3: All principles, issues and text examples in one table. [file 12910_2020_572_MOESM3_ESM.docx]

**S3 Table. Principles, issues and text examples.**

| **Principle/issue** | **Text example** | **Reference(s)** |
| --- | --- | --- |
| **Respect for participants** | | |
| Risk that legal protections fail to protect the dementia-related population because existing laws and policies do not apply to non-genetic test results from, e.g., amyloid biomarkers | „Unlike potential and similar risks associated with disclosing genetic markers, existing laws and policies that prohibit insurance or employment discrimination do not apply to nongenetic test results such as amyloid biomarkers. Additional gaps in legal protections fail to protect research subjects from discrimination by long-term care insurers, a particularly harmful risk to individuals whose disease foretells needing long-term care services.“ (Arias 2014) | (1–12) |
| Risk that there is a lack of guidance for professionals in risk-information disclosure, leading to harm | „A survey of Alzheimer’s Disease Neuroimaging Initiative (ADNI) researchers showed that both expert clinicians who supported and did not support disclosing amyloid imaging results recognized a need for a process to disclose safely and effectively and to study the impact of disclosure [8].“ (Harkins et al. 2015) | (7,10,13,14) |
| Risk that participants statements signify not a deliberate cognitive act but rather a means of engagement | “When patients spoke, they primarily agreed with and approved of what was said. Although at first this might seem to signal assent, for persons with dementia, such an interpretation should be made with caution. Because affirming statements may simply be a means of engagement rather than a deliberate cognitive act, it is difficult to assess the patient’s actual preference. That is, an apparent indication of assent may not be authentic. As such, this observation calls into question the rationale for suggestions that assent should be required for research with demented persons that is not directed toward personal benefit. 16,17 This has potentially important implications for efforts aimed at formulating approaches to geriatric assent, 18,19“ (Sugarman et al. 2007) | (3,12,15–17) |
| Challenge of imbalance between respecting participant autonomy and protection of the participant | “Respect for persons implies that we should not only respect prior autonomy, but also protect the no longer autonomous patients during the trial. We therefore need to find an adequate balance between respecting the prior autonomy of dementia patients and protecting the incompetent research participant during the trial. Dementia patients remain conscious and present during the trial, as opposed to many other situations advance directives are used for; i.e. post mortem directives. We should therefore remain cautious and look for signs of resistance or objection to undue burden during the research procedures.” (Jongsma et Vathorst 2015) | (2,3,5,7,8,18–31) |
| Risk of dependency of the participants on relationship with the researcher, which makes an after-trial transition plan/support necessary | “Sometimes, the person with dementia has become so deeply focused on the researcher that the researcher stepping back and withdrawing can only be achieved with the involvement and seamless stepping forward of another person.” (Dewing 2007) | (24,27,32,33) |
| Risk of harming dementia patient by disclosure of research results (e.g. reporting leading to harm, family disruptions by disclosure of risk information) | “Conducting preclinical AD clinical trials gives rise to a variety of novel ethical and policy challenges. These include whether to disclose genetic and/or biomarker results to an individual [...]” (Molinuevo et al. 2016) | (9,11,14,19,34–44) |
| Challenge of using adequate language, e.g., explicitly referring to the diagnosis as dementia or not, when communicating with the participant | “Hellstrom et al. (2007) decided not to use the word ‘dementia’ unless it was introduced by the person or their family, preferring the term ‘memory problem’. Bartlett and Martin (2002) discuss the potential deception of using this term and question whether fully informed consent is only possible when the person is fully aware of their diagnosis, but concomitantly, appreciating the harm and distress that may be evoked by a researcher unwittingly giving the person a diagnosis. Reid et al. (2001) faced a similar dilemma but concluded that it is important to meet the participants on their own terms and not insist on them admitting that they have dementia. The notion of active involvement in research rests on values of transparency, honesty and openness, perhaps then it is time to extend this to honesty about diagnosis.” (McKeown 2010) | (27,35,45,46) |
| Risk that the risk status is revealed by obvious side effects, if experimental drugs are given only to people at (high) risk | “One approach would be to test research participants for biomarkers that would indicate which participants would be at higher risk of having AD, or of having AD earlier, and give only those research participants promising experimental treatments. Participants who are found to be at lower risk would receive only a placebo drug. This approach would allow participants who are at lower risk to avoid any unknown side-effects of an experimental treatment, and only participants who are at higher risk for AD would risk unknown side-effects.  This approach makes sense, as participants who are at higher risk of having AD, or earlier AD, would stand to benefit more from receiving promising treatments, and those at lower risk for AD would not risk having unknown harmful side-effects. Researchers would not be able to tell which participants were at a higher risk because they would not know whether a participant was receiving a promising new drug or a placebo. (This can be achieved by having an out- side, third party assign participants to the various arms of a trial.) There is, though, possible additional risk for those who are at a higher risk of having AD or earlier AD. Participants who receive an experimental drug may be able to discern from its side- effects that they are the ones who are receiving the drug, and thus that they are at higher risk for AD or earlier AD. With this knowledge, some of the higher risk participants may feel despair.31”  (Howe 2015) | (47,48) |
| Risk that disclosure of risk information leading to harm of the study partner/(pre-)caregiver | “An individual who receives a preclinical AD diagnosis will learn something about his or her risk for progressing to dementia. The individual’s family members will learn something about their own risk, too: they will learn that they are at increased risk of needing to take on informal caregiving responsibilities for a loved one with dementia.” (Largent et al. 2018) | (37,50) |
| Risk that unexpected end of dementia trial leading to harm | “Occasionally, participants reflect how they see members of the study team more often than they see their own children or grandchildren. Thus, the unexpected end of a trial is the loss of a network of trusted and well-liked advisors. For some participants, it may be a sort of trauma, akin to a death or sudden and devastating illness.” (Largent et al. 2018) | (33) |
| Risk of lack of follow up plans for excluded participants at risk for developing dementia leading to serious harm, e.g. suicide | “Moreover, individuals found to be at risk for AD but disqualified for a trial for other reasons, such as an abnormal laboratory result, are excluded from the benefits of the group identity offered by trial participation [11]. This could exacerbate risks for suicide. Enrollment into an alternate research study or clinical followup may help minimize suicide risk and other poor outcomes.” (Stites 2018) | (42) |
| **Independent review** | | |
| Risk that research ethics committees’ (RECs) and/or IRB’s quality control of consent procedures are not uniform and comparable, e.g. in European countries and the U.S. | “The approval of a study by the responsible ethics committee should guarantee, amongst others, uniform quality control of consent procedures. Unfortunately, this uniformity does not exist in current research practice. Ethical reviewboards vary widely, both between and within countries, in the assessment of informed consent procedures [1].Moreover, in research in older participants, the consent assessment procedures are typically scarcely described, and detailed speciﬁcations are found in only very few papers [3]. In our survey of the literature [3], the frequency with which information on informed consent procedures by ethics committees were published was unexpectedly low” (Meulenbroek 2010) | (51–53) |
| Risk of RECs weighing opinions of physicians (protecting the participant) over patients’ willingness to participate and over nurse counsellors’ opinions | "The first amendment was interesting because it had been thought improbable that a consultant would override a person’s willingness to take part in the study and the judgement of a nurse counsellor. The consultant necessarily did this to protect the person from harm, but gatekeepers also support the development of a research culture in which people with dementia are helped to exercise their autonomy to its fullest potential. It is also noteworthy that the ethics committee accepted the consultant’s opinion of whether the person should participate in the research, rather than that of the nurse counsellor, a registered nurse working with people newly diagnosed with dementia." (Holland & Kydd 2015) | (21) |
| Risk that RECs systematically exclude patients with dementia because of different reservations, e.g. risks are too great; no other options than normal informed consent accepted | “Despite the growing research on participation, many researchers are required to sacrifice their values about participation by older persons with dementia when it comes to consent, often to satisfy the demands of research ethics committees (Grout, 2004). Ethics committees have a large amount of control in the continuum of exclusion and inclusion. Ethics committees are generally regarded by members of the academic and research community, including qualitative researchers, as running on bio-ethical principles; whether this is true or not. Consequently, in many situations, older persons with dementia do become excluded from being involved in research as active participants, as ethics committees may feel it is practically too difficult to do, the risks are too great and where informed consent is not applicable there have been no other detailed options set out for them (Dewing & Pritchard, 2004).” | (24,28,32,45,54–60) |
| **Fair participant selection/recruiting** |  |  |
| Risk of excluding relevant subgroups, e.g. inhabitants of nursing homes, those lacking a proxy/spouse or patients with other psychiatric diseases, from dementia research | “Despite the compelling individual and public health impact of nursing home care, clinical research, particularly clinical trials, rarely includes this population. The institutional characteristics of nursing home care may present challenges that deter investigators, or result in incomplete studies [7–10]” (Hanson et al. 2010) | (2,3,5,7,12,15,19,28–30,32,43,58–70) |
| Risk of excluding participants from research due to lack of capacity to consent | “At one extreme is the position that research studies should exclude all persons who lack the capacity to consent, a position that fails on two ethical counts. First, there is some subset of research studies that offer some prospect of benefit to participants; excluding persons who lack the capacity to consent may hinder the legitimate interests of those persons in improved clinical outcomes.” (Kim 2011) | (2,4,6–8,11,12,15,16,18,20,28,29,32,43,46,55,57,66,69,71–73) |
| Risk that informed consent (IC) is not valid using transparent enrollment (risk marker status-dependent inclusion) because its assessment is mandatory | “One major concern, expressed by investigators of blinded enrollment studies,7,8 is that transparent enrollment is coercive, presumably because persons who are not willing to learn their risk marker status are pressured to accept disclosure in order to have the opportunity to participate in a prevention trial. If this rationale is correct, then informed consent in a transparent design may not be valid.” (Kim et al. 2014) | (63) |
| Risk that gatekeepers in dementia research process hinder possible participants in participating in dementia research | “Bartlett and Martin (2002) note that gatekeepers may deny people with dementia the right to decide whether to participate in research, and recruiting people with dementia may depend on how gatekeepers perceive the research, gatekeepers’ relationships with the people with dementia, and the gatekeepers’ judgments about who should be involved in research.” (Lepore et al. 2017) | (28,29,46,74,75) |
| Risk that differing national legal frameworks on research leading to exclusion of people with dementia | “We are concerned, however, that differing legal frameworks across the country may restrict the types of research undertaken and inappropriately exclude people with dementia from participating in research. As a consequence, research may fail to tackle some of the more pressing issues, especially for people in later stages of dementia and those living in socially isolated circumstances. Research often excludes people with cognitive impairment (Taylor et al. 2012) because “many researchers and ethics committees are nervous about including this population in their studies” (Pachana et al. 2015, 705).” (Ries et al. 2017) | (60) |
| **Favorable risk-benefit-ratio** |  |  |
| Determining risk adequately |  |  |
| Risk of treating dementia patients unequally because there is no consensus on the definition of ‘minimal risk’ | "In practice there has been much disagreement among researchers and research ethics boards over what constitutes minimal risk. The threshold for minimal risk for a person with Alzheimer’s disease may be lower than that for someone else who is able to understand or tolerate a bone marrow biopsy, for example. Minimal risk is a relational judgment that is patient specific and context specific." (Slaughter 2007) | (12,53,76,77) |
| Risk of misconception of risk marker predictive value | “Second, employers may weigh the potential for decreased work product and potential safety issues. Education programs for employers related to the current knowledge connecting AD biomarkers to symptoms may resolve erroneous perceptions that an individual who has an AD biomarker will demonstrate symptoms that affect performance in the near future.” (Arias et al. 2014) | (1,10,11,13,23,34,35,38,78–80) |
| Risk of harm due to not-yet-known negative long term effects of disclosing the risk marker status | “Finally, transparent enrollment will allow researchers to study the effects of disclosure of risk marker status in a population broader than those who would desire disclosure at baseline, and will create data about people living with knowledge of being in an asymptomatic at-risk stage of a disease.” (Kim 2014) | (7,9–11,13,34,38,42,63,70,81) |
| Risk that risk marker-positive but asymptomatic people will take/receive off-label treatments, e.g. statins, or interventions in the hope of reducing their risk | “They desire to know if they are at risk for AD and, if they are, will take even unproven interventions to reduce this risk, and there are physicians willing to fulfill this desire.” (Karlawish 2011) | (22,79) |
| Risk of imbalance in risk-benefit ratio in adaptable trial designs leading to more benefits to later-accessing participants, which could lead to gaming the study by entering later | “Adaptive trials raise unique ethical challenges. Saxman21 identifies potentially troubling problems with equipoise, informed consent and justice. While it is always true that some participants in trials will benefit more than others (assuming any benefit at all), in conventional trials that difference is based on random selection. The first subject to enter the trial has the same chances for benefit as the 999th. In adaptive trials, which are constantly evolving, participants who delay their entrance into the trial are more likely to benefit. This may offer opportunities for sophisticated participants or their physicians to game the study by entering later.22 The evolving possibility of benefits presents one more difficulty in obtaining truly informed consent. Participants need to understand that the chance of being randomly assigned to one arm or another is based on accumulating data, not on simple chance, as in conventional trials. Of course, clinical trials can never hold out an expectation of benefit, but to the extent that the hope of benefit is an incentive for participants, they will need to understand that participants who enrol in the trial later may have a greater chance of benefit than earlier recruits.” (Davis 2017) | (61) |
| Considering risk adequately |  |  |
| Risk of over diagnosis in asymptomatic persons, if the diagnosis is derived from the risk marker status, since their corresponding validity regarding the occurrence and course of a disease is (still) limited | “This trend towards pre-symptomatic prediction has recently evoked strong ethical and sociological criticism (Le Couteur et al., 2013; Lock, 2013). Thus, David Le Couteur and his colleagues argue that it will mainly lead to overdiagnosis of AD and will therefore rather harm people.” (Schicktanz et al. 2014) | (11,35,38,41,42,82) |
| Risk of neglecting the psychological distress of asymptomatic persons caused by disclosure of the risk status | “There are also potential psychological risks involved in disclosing imminent risk information for a severe and incurable disease such as AD. Although the REVEAL data regarding psychological adjustment to test results are encouraging, group mean data may not apply to a given individual case; furthermore, the study involved mostly well-educated participants who were screened at baseline for suicidal ideation, and severe levels of depression and anxiety, limiting the generalizability of results [57]. Adverse psychological outcomes may be more likely if individuals misinterpret test results (e.g., taking an amyloid-positive result to mean that one is destined to develop AD), thus, underscoring the importance of patient education.” (Roberts et al. 2013) | (7,10,11,22,49,63,78,81) |
| Managing risks adequately |  |  |
| Risk of a lack of procedures to minimize harms of risk information disclosure | “As we discuss below, this ethical challenge means that prevention trials will need to develop careful procedures to minimize the harms that can accompany disclosure of risk information.” (Johnson 2015) | (7,38,49,83) |
| Risk of discrimination and/or stigmatization against relatives of participants in genetic risk research | “Respondents also mentioned the possibility of discrimination against relatives on the basis of results from genetic testing (for example by insurance companies).” (van der Vorm et al. 2008) | (84,85) |
| Risk of possible discrimination of risk marker positive participants | “An individual enrolled in a research study that uses positive test results from amyloid PET imaging or CSF measures of b-amyloid 42 as inclusion criteria has biomarkers indicative of AD pathology. If insurers and employers learn this information, it could expose subjects to discrimination.” (Arias et al. 2014) | (1,7,10,11,14,36,37,39,41,61,63,79,80,84–86) |
| Risk of inadvertent disclosure leading to harm, e.g. in blinded RCTs, in prospective cohort studies | “Research procedures are rarely flawless. Inadvertent disclosure of risk marker results could occur in a blinded enrollment design.” (Kim 2014) | (61,63,70,81) |
| Risk of non-disclosure of information to participants being unduly paternalistic | “Given these data, some would argue that withholding amyloid imaging results, particularly if obtained in the context of clinical research, could be unduly paternalistic.” (Roberts 2013) | (9,10,34,35,61,87) |
| Risk of participant stigmatization by the use of diagnostic labels such as dementia and mild cognitive impairment (MCI) | “Despite their clear benefits, diagnostic labels also serve as cues that activate stigma and stereotypes. Stigma associated with the diagnostic labels of dementia and mild cognitive impairment (MCI) can have a significant and negative impact on interpersonal relationships, interactions with the health care community, attitudes about service utilization, and participation in clinical research. The impact of stigma also extends to the family caregivers of individuals bearing such labels.” (Garand et al. 2009) | (7,11,14,19,22,26,27,37,47,85,88) |
| Risk of stigmatization by not using value-neutral and label-free language that is less likely to connote abnormality or foster a sense of “otherness” | “When individuals are reluctant to consider participating in studies due in part to stigma, investigators need to pay particular attention to the language used during recruitment encounters. For example, rather than continued use of the term “MCI,” we often use the phrase “changes in thinking” when referring to the condition. We are not suggesting that researchers or clinicians avoid providing information about the diagnosis of MCI. Rather, we have found that once a diagnosis is disclosed, there is a need to consider using value-neutral and label-free language that is less likely to connote abnormality or foster a sense of “otherness.” (Garand et al. 2009) | (7,9,20,25,27,45,46,88) |
| Risk of participant discrimination through insurers and employers gathering information about the risk of developing dementia | “An individual enrolled in a research study that uses positive test results from amyloid PET imaging or CSF measures of b-amyloid 42 as inclusion criteria has biomarkers indicative of AD pathology. If insurers and employers learn this information, it could expose subjects to discrimination.” (Arias et al. 2014) | (1,7,11,14,36–39,43,61,82,83,89) |
| Risk of risk marker status disclosure leading to misinterpretation of cognitive status and therefore harm | “Participants focused on how risk information could change their perception of their own cognition, leading to hypervigilance with regard to possibly dementia related symptoms: “it could be that if you ’ve been assessed as having a higher risk and then you ’ve gone a couple of days, and twice you ’ve come down without the thing you went up for . . . That could then make you think, ah, this is the beginning of it. ” (UK2) This was seen by some as a drawback of learning about one’s Alzheimer’s disease risk status. Moreover, this group further suggested that the revelation of dementia risk could affect their relationships with their families, including leading to their decisions or views being “second-guessed” (UK2).” (Milne et al. 2018) | (38,41) |
| **Social value** |  |  |
| Challenge of dealing with the uncertainty of a socially accepted wish to gain knowledge on dementia predisposition | “Is there a socially accepted wish to know or share knowledge about dementia predisposition? Is there social and cultural diversity about this? […]” (Schicktanz 2014) | (11,63) |
| Risk of interpreting findings from dementia research incorrectly because of poor reporting, especially details on informed consent | “Stocking et al. reviewed a sample of reports of trials including people with Alzheimer’s disease in 62 journals between January 1992 and December 1998.49 They found that, in 48% of the studies reviewed, no mention was made of involving participants in the consent and assent to participate in research process or of any individuals refusing or withdrawing. These authors suggested that editorial policy restricting article length could be a factor in the lack of information in this area.” (Slaughter 2007) | (12,90) |
| **Scientific validity** |  |  |
| Research design & planning |  |  |
| Challenge of balancing established standards against personal preferences for certain methodological considerations, e.g., limited number of eligible people in the dementia-related population, when a blinded enrollment is preferred | “However, investigators must in some situations use blinded enrollment in order to conduct a valid study. These situations are likely to occur only when the number of eligible subjects are limited and are part of an identifiable community whose values reflect a desire for blinded enrollment. The ethical basis for blinded enrollment in such a situation is the requirement of scientific validity, not the inherent value of the right not to know one’s risk marker status.” (Kim 2014) | (7,9,13,22,61,63,81,87) |
| Risk of dementia population fearing possible stigmatization, leading to low participation rates | “Dementia and MCI are diagnostic labels that have considerable use for health care providers and researchers in delineating specific patient populations that may benefit from clinical and research attention. However, these labels are associated with significant stigma that may affect individuals' willingness to seek and receive care, as well as participate in clinical research.” (Garand et al. 2009) | (7,47,88,91) |
| Risk of poor internal validity because of the heterogeneity of the MCI-population unless this is not compensated by recruiting more participants | “Trial design and evaluation are complicated because MCI is associated with a heterogeneity that seriously challenges the definition of a good study population. Given the annual reversion rates, it is difficult to assess whether an observed positive effect in a trial is due to the treatment tested or to therapy-independent reversion. This problem concerns the scientific value of MCI trials regarding the efficacy or internal validity, that is to say, reproducibility of results in a particular setting. Furthermore, it impacts on the purported medical value of the obtained study results in terms of their effectiveness or external validity.5 This is a particularly important issue for the application of research results in general practice, as I will lay out in the following. One strategy for dealing with the loss of power is to include more participants for a longer period of time. This alone challenges the internal validity of study results, however, for example, with regard to declining compliance and higher drop-out rates of study participants over time.” (Kutschenko 2011) | (78,92) |
| Risk of compromising external validity in high-risk research by including only participants capable of giving IC | “Second, the results raise some difficult questions for a policy that requires enrollment of only those determined to be competent to provide informed consent. Although it is possible that, given the high prevalence of AD, one could still recruit sufficient numbers for a small clinical trial--for example, involving sham neurosurgery to test a new intervention--it appears that recruiting unequivocally competent persons for such studies may prove difficult. At any rate, such subjects will represent a small, higher functioning subgroup of those who have AD, raising important issues concerning external validity; such subjects also have the most to lose from an adverse event, given their higher level of functioning.” (Kim et al. 2011) | (28,54) |
| Recruiting bias |  |  |
| Risk that transparent recruiting and the accompanying diagnostic label causes a smaller and less generalizable pool of potential participants | “Second, lack of willingness to be evaluated for a diagnostic label of dementia also means that investigators conducting research to ultimately improve the lives of affected individuals have a smaller and less generalizable pool of potential participants for clinical trials.” (Garand et al. 2009) | (88) |
| Risk of making high risk research, e.g., a neurosurgical gene transfer trial, impossible in late stage dementia if the consent of a competent person must be in direct chronological connection | “Suppose that, for a neurosurgical gene transfer trial for AD, it is decided that only patients who are competent to provide informed consent will be enrolled, owing to risk–benefit considerations. This is a theoretically reasonable approach but it relies on an empirically unproved assumption that it will be possible to enroll a sufficient number of patients with AD who are competent.” (Kim et al. 2011) | (6,16) |
| Risk of recruiting bias when competency to consent is an inclusion criterion leading to a non-representative sample of participants | “Furthermore, given that these unequivocally competent patients comprise a small and unusual subset of patients with AD, such patients may represent an atypical subgroup of those who have AD, raising issues concerning external validity.” (Kim et al. 2011) | (5–7,12,16,28,60,64,75) |
| Risk of undue exclusion of participants and jeopardized reproducibility of the study because of ambiguously formulated exclusion criteria that offer researchers too much freedom for selective recruiting | “A surprising finding that deserves attention is the frequent mention of ambiguous exclusion criteria. These criteria offer researchers too much freedom to selectively exclude potential research participants without the intervention of a Research Ethics Board. The selective exclusion of eligible research participants is, however, problematic for both scientific and ethical reasons.” (Jongsma et al 2016) | (64) |
| risk that the requirement of a study partner leads to low participation rates | “In a study of preclinical AD trial enrollment decisions, in which participants were randomly assigned to consider a hypothetical trial that did or did not require biomarker disclosure, we found that the study partner requirement was a more important barrier to enrollment when disclosure was required [12]. The requirement was rated as more important than drug risks in the disclosure arm of this study. These preliminary data suggest that preclinical AD trial participants may be reluctant to share with others that they have biomarker evidence of AD. Some participants may face an unenviable choice: have others potentially learn information about their health they do not want shared, or forego enrolling in a study in which they wish to participate.” (Grill & Karlawish 2017) | (70) |
| risk to delay scientific progress when studies fail to recruit adequate numbers of representative participants for AD studies | “Even in trials that eventually succeed in enrollment, participants rarely represent patients with AD on the whole given disparities in age, race, and comorbidities [6–8]. The failure to recruit sufficient numbers of representative participants at an acceptable rate delays scientific progress, wastes financial resources, and squanders the contributions that participants make to research [9].” (Grill & Karlawish 2017) | (93) |
| risk of generating a non representative sample by not including participants which lack a proxy | “The disproportionately high representation of spousal study partners is striking: individuals without a spouse or domestic partner comprise the majority of the population of potential research volunteers [21, 22].” (Largent et al. 2018) | (93) |
| Informant bias |  |  |
| Challenge of including underrepresented subgroups, especially persons living alone, without causing information bias, because here medical history is based on the statements of a person with dementia | “Enrollment of underrepresented populations, such as those lacking a spouse, in clinical trials must be increased. Such increases, however, may also necessitate developing methods to reduce informant bias, improve accuracy, and retain participants and study partners.” (Grill 2015) | (91,94,95) |
| Risk of risk information disclosure (e.g., at-risk status) leading to biased cognitive test results of the dementia-related participant (e.g. worsened test result because of negative self assessment) | “Similarly, participants’ perceptions of their expected performance on cognitive tests, which might be impacted by the return of test results, could bias subsequent outcomes (Hess et al., 2003; Steele and Aronson, 1995). Full consideration of these issues will require further study but may provide important data to instruct trial design and conduct.” (Grill et al. 2016) | (41,81,87,96) |
| Risk of getting inadequate information about the medical history important for research from a person diagnosed with dementia because of the cognitive decline | “Case–control studies present many problems, including different types of bias11 and the difficulties inherent in asking people in later life to remember what they ate and drank and did in the past. This is an important methodological problem for AD research, where some of the aetiology may reside decades before clinical onset, and especially since, having been identified as having dementia, subjects’ recall is automatically suspect.” (Davis 2017) | (61,95) |
| Risk that data provided by proxies differ from actual participants’ opinion | “Rather than include people with dementia in research, some studies include their proxies, such as caregivers or family members. However, data provided by proxies commonly differ from data provided by people with dementia (Clarke & Keady, 2002; Sands, Ferreira, Stewart, Brod, & Yaffe, 2004). For example, people with dementia have been found to have higher hopes for their quality of life than their caregivers do for them (Thorgrimsen et al., 2003).” (Largent et al. 2018) | (46) |
| Drop-outs |  |  |
| Risk that dementia patients experiencing stigmatization will lead to low follow-up rates or study withdrawal | “Stigma may have played a role in their decision to withdraw from the study, as these participants stated that they were uncomfortable with the questions asked during the initial assessment session.” (Garand et al. 2009) | (88) |
| risk that dementia prevention studies without participant study partners leading to higher dropouts leading to lower statistical power | “Study partners have key roles in assuring the validity of other aspects of preclinical AD trials. One role is minimizing missing data by preventing drop out. Preclinical AD trials are lengthy and participation can be burdensome, requiring many complex visits. Previous AD prevention trials have incurred greater than expected dropout [35], putting statistical power at risk.” (Grill & Karlawish 2017) | (70) |
| risk that non-spousal research dyads lead to lower completion rates in AD studies | “In AD trials, completion rates are lower among nonspousal dyads [10 ], and in one natural history study, informant replacement occurred more frequently in participants lacking a spouse [56 ]. Participant dropouts lower statistical power and create confounding if dropouts are non-random.” (Grill & Karlawish 2017) | (93) |
| Agenda setting |  |  |
| Risk of imbalanced research, because today, studies on dementia types with small prevalence are conducted more often than studies of other types | “In our study we found that the distribution of dementia research over the different types of dementia does not correspond with the prevalence of these dementia types in clinical practice.” (Jongsma et al 2016) | (64) |
| Risk of hindering international dementia research because implementations of EU-guidelines differ on a national level | “Firstly, implementation of the guidelines differs greatly across the EU countries, because of interaction with national legislation, resulting in hybrid procedures that may seriously hinder international multi-centre studies.” (Meulenbroek 2010) | (51,59) |
| **Collaborative partnership** |  |  |
| Risk of caregiver misrepresenting participants statements when they are consulted because of their knowledge of the participant | „Conversely, researchers must be cognizant that a caregiver could misrepresent the individual’s responses.“ (Black 2010) | (2) |
| Risk of lack of communication between researcher and possible dementia study population leading to selection | „This would have not only short-term consequences for the feasibility of recruiting from this pool of subjects but could also affect the long-term relationship between investigators and the community of at-risk persons. Without sufficient trust and collaboration of the community of eligible subjects, it would not be possible to conduct the research. Just as there is no right to participate in a research study (no subject can require that a researcher include him or her in a study), no researcher can compel anyone to participate in research. The only option is a mutually acceptable, voluntary agreement of cooperation. This highlights the importance of consulting the relevant communities during the early stages of trial design to determine their preferences. But the ethical principle that necessitates blinded enrollment in such a situation is, ultimately, scientific validity: a transparent design may not enroll sufficient numbers of subjects. The potential subjects’ desire not to know their risk marker status does not create, of itself, an obligation to use blinded enrollment.“ (Kim et al 2014) | (40,63,97) |
| Risk of reduced value of dementia research if different perspectives on dementia research are not taken into account | „Deliberative participatory processes in setting research and healthcare policy priorities should be strengthened to include the different perspectives and to benefit from the abundance of approaches.“ (Schicktanz 2014) | (11,26,97–99) |
| Risk of hindering public debate on dementia research because of a non-uniform language/communication | „In the framework of democratic regulation of research and healthcare, shifts in agenda and priority setting require public information and debate.“ (Schicktanz 2014) | (9,11,59) |
| Risk that professional guidelines are not based on a broad empirical background, especially concerning lay people dealing with dementia | „Professional guidelines should be based on more empirical, cross-cultural studies on how lay people actually deal with LOAD risk prediction.“ (Schicktanz 2014) | (10,11,60,99) |
| **Informed consent (IC)** |  |  |
| Qualified personnel |  |  |
| Risk that less-experienced researchers will lack the skills necessary for a sensible and adequate handling of the challenges that appear in the informed consent process in research with dementia patients | „Researchers begin social engagement with the person prior to gaining initial consent. In some settings this may take several visits or weeks. Thus the researcher has already collected information about the person, either from others or though their own observations and interactions to begin the process of assessing their capacity. The method may appear limited given the requirement of time and perhaps the high level of person-centred expertise including communication skills required from researchers. The ability to be reflexive in the field is central to this method, and this may be challenging for novice researchers to achieve, especially if not combined with effective coaching from more experienced colleagues. Thus the method may not be suitable for use by less experienced researchers or those working in isolation.“ (Dewing 2007) | (4,20,32,55) |
| Good guidance |  |  |
| Risk of overgeneralization of specific problems and low focus on dementia patients because of a lack of dementia specific guidelines on IC issues | „We selected eight influential legal and ethical documents that set rules or guidelines for conducting biomedical research with human subjects, and have focused on the articles that refer to subjects who are not able to provide informed consent. None of the guidelines state specific rules for dementia patients. They are included in the group of incapacitated adults, which also includes for example mentally disabled persons and persons in a coma.“ (Jongsma et al. 2015) | (29,30,43,57,73,100,101) |
| Right (amount of) information |  |  |
| Risk of uncertainty about what to disclose to the participant because there is no clear guidance on what risk information should be disclosed to the dementia patient | The prospect of disclosing amyloid imaging results and related AD risk information to asymptomatic individuals raises numerous challenges, necessitating in-depth empirical research and consideration of ethical dilemmas.” (Roberts 2013) | (10,11,34,38,40,42,49,87) |
| risk of undermined IC because of lack of information on efficacy and safety in deep brain stimulation studies | “Ethical issues in DBS for AD research with human subjects […] Risk of undermined informed consent: Lack of information on efficacy and safety: Are there concordant, valid and disease-specific preclinical findings replicated in different animal models and species?” (Bittlinger & Müller 2018) | (92) |
| Challenge of balancing the intention not to harm participants by using stigmatizing diagnostic labels (such as dementia or MCI) and IC being not valid in such cases because of lack of information | “Concealing the dementia focus of a project and avoiding the term ‘dementia’ may protect participants from distress but it also raises several issues that warrant consideration. First, it can be argued that this approach constitutes deception and violates the principle of fully informed consent (Bartlett & Martin, 2002). Researchers who use terms like ‘memory problems’ during the course of research should consider how they plan to report their findings. Avoiding the term dementia during the research process, but then using it in presentations or publications could be viewed as deceptive and potentially harmful.” (Novek & Wilkinson 2019) | (74) |
| Understanding |  |  |
| Risk of therapeutic misconception of pre-symptomatic dementia patients (e.g. biomarker positive or pet-ct positive populations) | “Out of fear, pre-symptomatic persons may also be more vulnerable to therapeutic misconception (Fisher et al., 2012) and might falsely expect a therapeutic benefit of biomarker research.” (Schicktanz 2014) | (3,5,11,14,35,40,73,86,91,94) |
| Risk of therapeutic misconception of dementia patients applying for deep brain stimulation studies | “Ethical issues in DBS for AD research with human subjects […]Risk of therapeutic misconception: Regulation: Are patients aware of the research context or is it masked as therapeutic “compassionate use” or “clinical innovation” instead of nontherapeutic research e.g. under FDA’s Investigational Device Exemption label?” (Bittlinger & Müller 2018) | (92,102) |
| Risk of therapeutic misconception being higher in participants with MCI or mild dementia | “Therefore, even in people with MCI or mild dementia, the ability to understand the distinctions between research and clinical care and how these distinctions may affect one’s own well-being in a clinical trial may be cognitively out of reach.” (Dunn & Palmer 2017) | (52) |
| Capacity assessment |  |  |
| Risk that capabilities of participants will be overestimated, especially if the patients are not yet completely incompetent | “The main difficulty lies in those who are in the gray area of mental competence, and this region is quite broad. Even expert clinicians frequently err in their evaluations, usually towards assessing patients to be more competent than they actually are.” (Korczyn 2007) | (69,103,104) |
| Risk that cognitive assessment tests (e.g. MMSE) in dementia research are harmful because they focus on people’s deficits rather than their strengths | “The one notable exception to this concerned the use of the MMSE. Originally this was intended to provide a more ‘objective’ indicator of cognitive capacity. However, as with Pratt,26 we found that the MMSE score bore no relationship to the ability of persons with dementia to talk about their experiences, and, rather than prove helpful, it was potentially detrimental on a number of fronts. The incident about the woman who refused to complete the MMSE after her recent assessment of her continuing capacity to drive was not an isolated one. The MMSE focuses on people’s deficits rather than their strengths and can be a blow to their self-esteem and a potential threat to their ‘dignity as identity’.15 One person with dementia, when asked to write a spontaneous sentence as part of the MMSE, wrote: ‘I’m stupid.’ When asked why she had done this she explained that it was because this was how the test made her feel. Quite the reverse happened in another interview when a person with dementia who had undergone several years’ academic education, and had formerly been a senior businessman, found the MMSE insulting and stated that ‘the test has an air of ridicule’. We would strongly recommend that careful consideration is given to the appropriate use of the MMSE in future research of this type. Considerable effort was invested in ensuring that positive relationships were created and sustained for both parties, but especially the persons with dementia. This itself raises ethical questions about the morality of relationship building over time when eventually the research relationship will be terminated. Once again there are no easily or universally correct solutions, but we believe that if handled with sincerity and honesty the overall experience can be very positive for all involved." (Hellström et al. 2007) | (20,27,28,69,95) |
| Risk of confusing the expressed willingness of a dementia patient to participate in research with the capacity to consent | “One intriguing speculation is an inadvertent conflation of capacity with authenticity: even a considerably impaired patient with reduced capacity for informed consent may be able to convey a sense of his or her genuine (that is, authentic) willingness to participate in research. This expressed willingness can, understandably, be mistaken for a sign of intact capacity (Box 1).” (Kim 2011) | (16,18) |
| Risk of taking a diagnosis of dementia as an exclusion criterion without considering the actual competency of the patient | “These and other studies demonstrate that a diagnosis of mild to moderate Alzheimer’s cannot be used as an automatic judgment of incapacity to consent to research. Instead, the investigator must look at the patient’s severity of overall cognitive impairment and assess that patient’s decisional capacity.” (Johnson et al. 2015) | (7,15,19–21,26,27,43,46,105) |
| Risk that cognitive assessment tests in dementia research lack a final determination of capacity | “One of the limitations of existing instruments, including the MacCAT-T and MacCAT-CR, is the lack of a final determination of whether someone has or lacks sufficient capacity to provide informed consent [9].” (Dunn 2009) | (4,8,16,19,32,43,51,66,89,90,105,106) |
| Obtaining IC (incl. safeguards) |  |  |
| Risk that the obligation of proxy consent in dementia research slows down the recruitment process and can endanger scientific validity | “The requirement that informed consent be given by a legally authorised representative dramatically slowed down the recruitment process in AdCare.” (Gainotti 2010) | (6,57,93) |
| Risk of misjudging the actual meaning of a patients expression of dissent regarding study participation | “Participants agreed that researchers should respect an individual’s objection whenever it occurs by not initiating the study or study procedure (or it may occur during a study procedure) and then immediately assessing its meaning. Determining what expressions or indications are truly reflective of dissent can be challenging in persons with dementia and requires the judgment of a person with expertise in dementia.” (Black 2010) | (2,12) |
| Risk of excluding dementia patients because of a too-rigid study design that could not wait for a "good day" to include a person | “Timing was particularly important, in the case of one participant who had very few ‘good days’ where he was able to communicate, the researcher needed to be able to seize the opportunity to meet with him on a ‘good day’. […] Hubbard et al. (2003) reflect that their research design did not provide the flexibility required to respond to the individual’s needs to communicate, for example, a participant might be more communicative on a day the researcher was visiting another person, and the researchers were unable to respond because of a rigid protocol. The need for such flexibility is supported by McKillop and Wilkinson (2004) who urge researchers to recognise if a person is becoming tired and re-schedule a further visit to complete the interview.” (McKeown 2010) | (45) |
| risk of IC process in first in human studies is invalid because of vulnerability of research participants | “To make a well-informed decision, potential research subjects do not only need to know, among other things, their subjective interests and risk preferences. A well-informed decision requires also some cognitive access by research subjects such as Ms. Metis to what would be in her best interest from a more objective point of view. That is, independent of how Ms. Metis evaluates the facts, she must have some understanding of the facts . However, to know what is in one’s own best interest in an epistemically complex situation as outlined may go well beyond one’s cognitive agency. This is hampered by external influence such as time pressure, urgency, or affective states such as fear and anxiety or even mild cognitive impairments. Moreover, the degree of desperation constitutes a vulnerability of people diagnosed with a severe medical condition for which no effective therapy is yet available. This is “vulnerability” in a nonstigmatizing sense, because it does not devalue the rights of these persons to make their own decisions, given legal capacity.” (Bittlinger 2018) | (102) |
| Risk of IC being insufficient to safeguard confidentiality in regard to big data approaches in dementia research | “Moreover, informed consent—in its current shape—does not grant data subjects (nor their legal representatives) sufficient control over highly sensible information regarding their cognitive state. This is a disincentive for people to make their data available for research in the first place.” (Ienca et al. 2018) | (31,82) |
| Risk of conflict of interest if researcher has the decision-making authority over the participant | “People who live alone, are distant from family members, or have dysfunctional families may be excluded from research. As one Australian researcher said in a recent survey: It is very difficult to conduct research with older people who do not have a carer or family member who can give consent in addition to the older person. As a result, older people who do not have a carer tend to not be included in the sample.(Pachana et al. 2015, 704) The Victorian legislation deals with this problem by allowing a registered practitioner to carry out or supervise the carrying out of a medical research procedure on a person who does not have capacity to give consent and does not have a person responsible (Guardianship and Administration Act 1986 (Vic) s 42S). While this provision allows researchers to overcome the need to resort to a tribunal, it has been criticized for creating a conflict of interest by inappropriately delegating decision-making authority to researchers (Victorian Law Reform Commission 2012).” (Ries et al. 2017) | (29,60,75) |
| Proxy consent |  |  |
| Risk that in dementia research proxy feels unable to decide if no written advanced research directive (ARD) exists | “Many caregivers were hesitant about moving forward without some type of written document from the loved one or some legal appointee, at a point in their life where they were capable of expressing their wishes.” (De Vries 2010) | (5–7,12,18) |
| Challenge of balancing divergent statements in ARD against current dementia patient wishes or proxy decisions (now vs. then) | “Over one-quarter of the general population whose current wish is not to participate in future AD research also say that they would be willing to allow their future surrogates the leeway, if and when the times comes, to enroll them.39 Among people who have first-degree relatives with AD, 80% state that their families could enroll them in potentially beneficial research even when their advance directive opposes enrollment in research.32 This is not a paradox. Such people may be aware that their current preferences are speculative and thus value them less than their trust in the judgments of their loved ones who may have a more complete set of facts in the future.” (Kim 2011) | (2–8,11,12,15,16,19,20,29,31,43,47,53,59,65,73,107) |
| Risk of focusing only on proxy in consent process and neglecting the person with dementia | “Post suggests that current approaches, which do not necessarily require contact with the person with dementia until after proxy consent has been given, amounts to exclusionary ethics.39” (Slaughter 2007) | (8,12,15,18,20,45,53,66,70,92) |
| Risk that proxy consent is limited to therapeutic research and non-therapeutic research with minimal risk and minimal burden | “Thirdly, the possibilities of doing research without the consent of the research participant are rather limited. Proxy consent is a less robust authorization than authorization by the research subject himself, and consent by a legal representative has only little moral authority compared to autonomous authorization by the research subject. Therefore, the measure and extent to which a third party may expose the incompetent research subject to risk or harm is limited to either therapeutic research, or non-therapeutic research with minimal risk and minimal burden (e.g. Biomedicine Convention 1997; European Clinical Trials Directive 2001). The possibilities of doing research with dementia patients, based on proxy consent, are therefore limited.” (Jongsma et Vathorst 2015) | (6–8,11,12,16,18,53,57,61,65,66) |
| Risk that varying (inter)national regulations are a burden for (inter)national dementia research | “However, only in Germany and Italy the system of proxy is determined by the courts - a procedure which is not necessarily required for the recognition of a proxy in other member states.” (Gainotti 2010) | (4–7,12,18,29,31,43,61,72,76) |
| Risk that proxy consent in dementia research becomes more difficult to achieve with increasing numbers of possible proxies in a family | "In fact, in our study group the number of sons and daughters was a predictive variable of ‘‘non appointment’’ of the legal proxy. The more cited reasons for not appointing a legal proxy were the impossibility to achieve an agreement among relatives, followed by the relatives’ fear that the legal proxy may take advantage of his position.” (Gainotti et al. 2010) | (6) |
| Risk of not considering that proxies have major self interest in dementia research, e.g., because they have same genetic traits, which could influence their proxy decision, and their manipulative behavior may be difficult to detect | “Proxy consent, already an issue of debate in traditional research, was considered more problematic in genetic research, where children share the same genetic traits as their parents. On the one hand, this might be a motivation for the affected parent to participate in a research study to help their children. On the other hand, it was questioned that to what extent children still are able to make a decision in the best interest of their parents because they have an interest themselves. The more genetic research will be carried out, the higher the chance on a disease modifying or preventive therapy for them and their children.” (Olde Rikkert et al. 2008) | (4–6,12,20,29,31,43,51,55,84,85,93,108) |
| Risk that proxy of a dementia patient misunderstands aspects of research, e.g. therapeutic misconception | “In several studies, many proxies hoped for direct benefits, even from non-direct benefit research or those with very low probabilities of benefit [52,54]—even when explicitly told the study did not carry potential for direct benefit [14].” (Dunn 2009) | (4,43,52) |
| Risk of proxy consent in dementia research being a moral burden for legal representative | “Secondly, legal representatives experience their task to make decisions for their incompetent loved ones’ as difficult, and have problems in bearing the burden and responsibility of making decisions for a dementia patient (Livingston 2010; Sugarman et al. 2001). They experience guilt and stress and have problems with processing the provided information (Wendler and Rid 2011). To make decisions as a legal representative is especially hard in circumstances in which long time roles and patterns of authority are reversed and confidences are sometimes breached (Livingston et al. 2010). For example, it is conceivable that a child who has always been obedient to his parents, will have difficulties in taking the lead when his authoritarian parent becomes incompetent to make his own decisions.” (Jongsma et Vathorst 2015) | (4–8,12,18,45,60,71,108,109) |
| Risk that proxy consent in genetic dementia research in larger families might violate the right-not-to-know of individuals if every family member is not included in the consent process | “One of the most important issues mentioned during the focus group meeting was family consent in genetic research. Family consent was considered an alternative for asking individual consent of a number of family members. Although family consent might raise new practical problems, it was suggested that this possibility should be analysed in more detail because every relative of a person being tested for AD is a stakeholder. Family consent is discussed in the literature, but the meaning of the term varies with context.” (van der Vorm et al. 2008 – Genetic | (84,85) |
| risk of not fulfilling the obligation of IC by only obtaining proxy consent in first-in-human studies | “Finally, our ethical analysis revealed that at least in some cases informed consent was reported to be obtained from surrogates only. In our opinion this would raise serious concerns. In agreement with German law and the Declaration of Helsinki,20 we think that informed consent of each and every participant is indispensable for investigational first-in-human research on risky neurosurgical interventions such as DBS with presently unproven direct therapeutic benefit to participants.” (Bittlinger & Müller 2018) | (92) |
| Broad consent |  |  |
| Risk that broad consent leading to harm, e.g. privacy issues concerning inadequate data use | “Broad consent does raise concerns about privacy and informational autonomy and tensions with data protection requirements in some jurisdictions [28,29].” (Thorogood et al. 2017) | (43) |
| Advance research directives (ARD) |  |  |
| Risk of confusion concerning ARD issues because of no existing guidelines towards ARDs in dementia research | “ARDs for dementia research have been mentioned as early as 1998,31 but there is still no national legislation or official guidelines governing their use.30 Perhaps an organization involving many stakeholders, such as the Alzheimer’s Association, could lead the way in such an endeavour. Considerations would include appropriate wording; the timing of required renewals; whether ARDs could allow individuals to consent to research that is higher than minimal risk; whether family members or designated proxies could veto participation in research.” (Davis 2017) | (29,43,53,59–61,65,107) |
| Risk that ARD in dementia research cannot be a truly informed decision because of the impossibility of anticipating a situation never experienced | “Dresser (1999) states that an anticipated decision cannot be a truly informed decision, because the competent person needs to anticipate a situation he has never experienced; namely being incapacitated.” (Jongsma et Vathorst 2015) | (3,4,7,8,12,15,61,65) |
| Risk that new information (“body of evidence”) presents an obstacle to the interpretation of ARDs in dementia research | “The sufficiency of information can be questioned when during the time gap between signing and the use of the ARD, new information about the research trial or about specific procedures emerges.” (Jongsma et Vathorst 2015) | (3,4,8,29,43,65) |
| Risk that ARDs have no practical relevance - popular in theory but not used in high numbers in dementia research | “Although advance directives are mentioned in most policy discussions, the rates of completion of research advance directives are likely to remain low.31,32” (Kim 2011) | (5,7,16,43,54,60,65,67,71,107,110) |
| Ongoing assessment |  |  |
| Risk that IC at the beginning of a dementia study alone is insufficient because of cognitive decline of participants | “However, a special ethical issue with regard to longitudinal studies that end in participants’ death is that participants are competent when first recruited, but have a significant likelihood of becoming incompetent while they are study subjects. ‘[O]ne of the primary challenges to conducting research on dementia involving this population is the gradual loss of the capacity to consent’.14 This creates challenges for informed consent, the ethical bedrock of research with human subjects. ‘In cases where a subject’s cognitive condition is expected to deteriorate or fluctuate, it may make sense to re-evaluate consent capacity … at several intervals during the study, especially in long-term studies that may involve multiple phases’." (Davis 2017) | (20,29,43,61,103) |
| Challenge of dealing with varying standards/thresholds to determine and re-evaluate competency/capacity | “Competence to consent to either treatment or research participation is not a simple matter of the stage of dementia or severity of cognitive impairment for an individual. In part, this is because competence is not a unitary construct. One can be competent in some aspects of one’s life (eg, competent to consent to research) without being competent in others (eg, competent to drive a motor vehicle) [24]. As the risk/benefit ratio and other aspects of a given treatment or study vary, so do the requirements for competence.” (Fisk 2007) | (4,5,8,11,12,16,19,29,43,54,57,66,103,105) |
| Risk of not monitoring signs of distress throughout data collection to satisfy the core idea of IC, especially if no standard IC procedure was possible because of the participants dementia | “Although the standard approach to informed consent does not require any processes for seeking and maintaining assent from the person with dementia, several have recommended that signs of distress be monitored throughout data collection.6,14,19,25,47” (Slaughter 2007) | (12,100) |
| risk of not being able to differentiate dissent from symptoms of dementia | “This has the potential drawback of delaying or preventing research to understand and treat disorders such as agitation, since it can be difficult to discern if the behavior is an indication of dissent or a symptom of the disease that needs to be treated and for persons with severe cognitive impairment in whom the ability to clearly state a willingness to participate may be impaired.” (Black et al. 2010) | (2,108) |
| Risk that possible ‘direct benefit considerations’ will lead to overstepping the ‘right to dissent at any time’ in a dementia study | “On the question of whether the dissent of an individual to participate in research should ever be overridden if the study provides the potential for a direct benefit available only in research, findings from this study differ from the positions taken by some previous groups (8,11,21, 22). The national panel concluded that superseding fundamental ethical principles and overriding dissent is unjustified and unnecessary. That is, best interest does not override dissent, even when a person cannot understand the potential benefit of research.” (Black et al. 2010) | (2,12,46,53,108) |
| Informed consent document (ICD) |  |  |
| Risk that simplified IC forms will lead to psychological distress for dementia patients | “Some persons with dementia can be reasonably expected to participate in full informed consent with alterations made for the consequences of the early changes in cognition and have a meaningful, informed and engaged experience for both parties. Offering simplified information and consent forms is however only one way forward and can be a source of anxiety for some [25].” (Dewing 2008) | (15,19) |
| Ethical oversight |  |  |
| Risk of insufficiently informing IRBs about adequate steps taken to fulfill the ethical obligations of dementia research | “The range of remaining cognitive abilities of adults who lack consent capacity, the presence of neuropsychiatric symptoms, and changes in the individual’s temperament pose challenges when seeking assent and respecting dissent. Judgments must be based on the researcher’s observation of that individual and the situation at hand, a rigorous evaluation of the person’s abilities, probing to understand what an expression or indication really means, and addressing when possible any unrelated distress or concerns of the individual. Explicit discussion of these issues when an inability to consent is likely might reassure IRBs that adequate steps are being taken to fulfill the ethical obligations inherent in conducting dementia research.” (Black et al. 2010) | (2,85) |
| Risk that uncertainties about consent in dementia research will lead to problems in the participation in longitudinal studies | “As highlighted in the 2015OECDBigData for Dementia report, uncertainties about consent issues in the context of dementia research may undermine participation in longitudinal studies.” (Thorogood et al. 2017) | (31) |

**References**

1. Arias JJ, Karlawish J. Confidentiality in preclinical Alzheimer disease studies: When research and medical records meet. Neurology. 2014 Feb 25;82(8):725–9.

2. Black BS, Rabins PV, Sugarman J, Karlawish JH. Seeking Assent and Respecting Dissent in Dementia Research. Am J Geriatr Psychiatry. 2010 Jan;18(1):77–85.

3. Buller T. Advance consent, critical interests and dementia research. J Med Ethics. 2015 Aug;41(8):701–7.

4. Dunn LB, Misra S. Research Ethics Issues in Geriatric Psychiatry. Psychiatr Clin North Am. 2009 Jun;32(2):395–411.

5. Fisk JD, Beattie BL, Donnelly M. Ethical considerations for decision making for treatment and research participation. Alzheimers Dement. 2007;3(4):411–7.

6. Gainotti S, Imperatori SF, Spila-Alegiani S, Maggiore L, Galeotti F, Vanacore N, et al. How are the interests of incapacitated research participants protected through legislation? An Italian study on legal agency for dementia patients. PLoS One. 2010;5(6):e11150.

7. Johnson RA, Karlawish J. A review of ethical issues in dementia. Int Psychogeriatr. 2015 Oct;27(10):1635–47.

8. Jongsma KR, van de Vathorst S. Beyond competence: advance directives in dementia research. Monash Bioeth Rev. 2015 Sep;33(2-3):167–80.

9. Molinuevo JL, Cami J, Carné X, Carrillo MC, Georges J, Isaac MB, et al. Ethical challenges in preclinical Alzheimer’s disease observational studies and trials: Results of the Barcelona summit. Alzheimers Dement [Internet]. 2016 Mar [cited 2016 Apr 21]; Available from: http://linkinghub.elsevier.com/retrieve/pii/S1552526016000765

10. Roberts JS, Dunn LB, Rabinovici GD. Amyloid imaging, risk disclosure and Alzheimer’s disease: ethical and practical issues. Neurodegener Dis Manag. 2013 Jun;3(3):219–29.

11. Schicktanz S, Schweda M, Ballenger JF, Fox PJ, Halpern J, Kramer JH, et al. Before it is too late: professional responsibilities in late-onset Alzheimer’s research and pre-symptomatic prediction. Front Hum Neurosci. 2014;8:921.

12. Slaughter S, Cole D, Jennings E, Reimer MA. Consent and assent to participate in research from people with dementia. Nurs Ethics. 2007;14(1):27–40.

13. Harkins K, Sankar P, Sperling R, Grill JD, Green RC, Johnson KA, et al. Development of a process to disclose amyloid imaging results to cognitively normal older adult research participants. Alzheimers Res Ther [Internet]. 2015 Dec [cited 2017 Jan 6];7(1). Available from: http://alzres.com/content/7/1/26

14. Alpinar-Sencan Z, S S. Addressing ethical challenges of disclosure in dementia prediction: limitations of current guidelines and suggestions to proceed [Internet]. BMC medical ethics. 2020 [cited 2020 Sep 21]. Available from: https://pubmed.ncbi.nlm.nih.gov/32393330/

15. Dewing J. Process consent and research with older persons living with dementia. Res Ethics Rev. 2008;4(2):59–64.

16. Kim SYH. The ethics of informed consent in Alzheimer disease research. Nat Rev Neurol. 2011 May 24;7(7):410–4.

17. Sugarman J, Roter D, Cain C, Wallace R, Schmechel D, Welsh-Bohmer KA. Proxies and Consent Discussions for Dementia Research: PROXY CONSENT. J Am Geriatr Soc. 2007 Apr;55(4):556–61.

18. De Vries R, Stanczyk A, Wall IF, Uhlmann R, Damschroder LJ, Kim SY. Assessing the quality of democratic deliberation: a case study of public deliberation on the ethics of surrogate consent for research. Soc Sci Med 1982. 2010 Jun;70(12):1896–903.

19. Heggestad AKT, Nortvedt P, Slettebø \AAshild. The importance of moral sensitivity when including persons with dementia in qualitative research. Nurs Ethics. 2012;0969733012455564.

20. Hellström I, Nolan M, Nordenfelt L, Lundh U. Ethical and Methodological Issues in Interviewing Persons With Dementia. Nurs Ethics. 2007 Sep 1;14(5):608–19.

21. Holland S, Kydd A. Ethical issues when involving people newly diagnosed with dementia in research. Nurse Res. 2015 Mar 18;22(4):25–9.

22. Karlawish J. Addressing the ethical, policy, and social challenges of preclinical Alzheimer disease. Neurology. 2011 Oct 11;77(15):1487–93.

23. Lingler JH, Klunk WE. Disclosure of amyloid imaging results to research participants: Has the time come? Alzheimers Dement. 2013 Nov;9(6):741–4.e2.

24. Hodge J, Foley S, Brankaert R, Kenning G, Lazar A, Boger J, et al. Relational, Flexible, Everyday: Learning from Ethics in Dementia Research. In: Proceedings of the 2020 CHI Conference on Human Factors in Computing Systems. 2020. p. 1–16.

25. Jongsma K, M S. Return to childhood? Against the infantilization of people with dementia [Internet]. Bioethics. 2018 [cited 2020 Sep 21]. Available from: https://pubmed.ncbi.nlm.nih.gov/30106171/

26. Mann J, Hung L. Co-research with people living with dementia for change. Action Res. 2019;17(4):573–90.

27. Novek S, H W. Safe and Inclusive Research Practices for Qualitative Research Involving People with Dementia: A Review of Key Issues and Strategies [Internet]. Dementia (London, England). 2019 [cited 2020 Sep 21]. Available from: https://pubmed.ncbi.nlm.nih.gov/28350179/

28. Ries Nm, M T. Bioethics and Universal Vulnerability: Exploring the Ethics and Practices of Research Participation [Internet]. Medical law review. 2020 [cited 2020 Sep 21]. Available from: https://pubmed.ncbi.nlm.nih.gov/32524142/

29. Thorogood A, Dalpe G, McLauchlan D, Knoppers B. Canadian Consent and Capacity Regulation: Undermining Dementia Research and Human Rights. McGill J Law Health. 2018;12:67.

30. Thorogood A, A M-P-L, G D, C G, S G. Openness, inclusion, and respect in dementia research [Internet]. The Lancet. Neurology. 2019 [cited 2020 Sep 21]. Available from: https://pubmed.ncbi.nlm.nih.gov/30663603/

31. Thorogood A, C DS-P, Bm K. Substitute consent to data sharing: a way forward for international dementia research? [Internet]. Journal of law and the biosciences. 2017 [cited 2020 Sep 21]. Available from: https://pubmed.ncbi.nlm.nih.gov/28852560/

32. Dewing J. Participatory research A method for process consent with persons who have dementia. Dementia. 2007;6(1):11–25.

33. Largent Ea, J K. Rescuing Research Participants After Alzheimer Trials Stop Early: Sending Out an SOS [Internet]. JAMA neurology. 2020 [cited 2020 Sep 21]. Available from: https://pubmed.ncbi.nlm.nih.gov/32011643/

34. Bunnik Em, E R, R M, Mhn S. On the personal utility of Alzheimer’s disease-related biomarker testing in the research context [Internet]. Journal of medical ethics. 2018 [cited 2020 Sep 21]. Available from: https://pubmed.ncbi.nlm.nih.gov/30154216/

35. Erdmann P, M L. The Ambivalence of Early Diagnosis - Returning Results in Current Alzheimer Research [Internet]. Current Alzheimer research. 2018 [cited 2020 Sep 21]. Available from: https://pubmed.ncbi.nlm.nih.gov/28891443/

36. Kim H, Jh L. Disclosure of amyloid PET scan results: A systematic review [Internet]. Progress in molecular biology and translational science. 2019 [cited 2020 Sep 21]. Available from: https://pubmed.ncbi.nlm.nih.gov/31481171/

37. Largent Ea, K H, Ch van D, S H, P S, J K. Cognitively unimpaired adults’ reactions to disclosure of amyloid PET scan results [Internet]. PloS one. 2020 [cited 2020 Sep 21]. Available from: https://pubmed.ncbi.nlm.nih.gov/32053667/

38. Milne R, E B, A D, E R, S B, D G, et al. Perspectives on Communicating Biomarker-Based Assessments of Alzheimer’s Disease to Cognitively Healthy Individuals [Internet]. Journal of Alzheimer’s disease : JAD. 2018 [cited 2020 Sep 21]. Available from: https://pubmed.ncbi.nlm.nih.gov/29480179/

39. Milne R, J K. Expanding engagement with the ethical implications of changing definitions of Alzheimer’s disease [Internet]. The lancet. Psychiatry. 2017 [cited 2020 Sep 21]. Available from: https://pubmed.ncbi.nlm.nih.gov/28347435/

40. Robillard JM, Feng TL. When patient engagement and research ethics collide: lessons from a dementia forum. J Alzheimers Dis. 2017;59(1):1–10.

41. Schermer Mhn, E R. On the reconceptualization of Alzheimer’s disease [Internet]. Bioethics. 2019 [cited 2020 Sep 21]. Available from: https://pubmed.ncbi.nlm.nih.gov/30303259/

42. Stites Sd. Cognitively Healthy Individuals Want to Know Their Risk for Alzheimer’s Disease: What Should We Do? [Internet]. Journal of Alzheimer’s disease : JAD. 2018 [cited 2020 Sep 21]. Available from: https://pubmed.ncbi.nlm.nih.gov/29480203/

43. Thorogood A, Mäki-Petäjä-Leinonen A, Brodaty H, Dalpé G, Gastmans C, Gauthier S, et al. Consent recommendations for research and international data sharing involving persons with dementia. Alzheimers Dement. 2018 Oct 1;14(10):1334–43.

44. Grill Jd, Cg C, K H, J K. Reactions to learning a “not elevated” amyloid PET result in a preclinical Alzheimer’s disease trial [Internet]. Alzheimer’s research & therapy. 2018 [cited 2020 Sep 21]. Available from: https://pubmed.ncbi.nlm.nih.gov/30579361/

45. McKeown J, Clarke A, Ingleton C, Repper J. Actively involving people with dementia in qualitative research. J Clin Nurs. 2010 Jul 1;19(13-14):1935–43.

46. Lepore M, Shuman SB, Wiener JM, Gould E. Challenges in involving people with dementia as study participants in research on care and services. Res Summit Om Dement Care Build Evid Serv Supports. 2017;

47. Howe E. Mediation Approaches at the Beginning or End of Life. - PubMed - NCBI. J Clin Ethics. 2015;26(4):275–85.

48. Hooper M, Grill JD, Rodriguez-Agudelo Y, Medina LD, Fox M, Alvarez-Retuerto AI, et al. The impact of the availability of prevention studies on the desire to undergo predictive testing in persons at risk for autosomal dominant Alzheimer’s disease. Contemp Clin Trials. 2013;36(1):256–62.

49. Janssens Acj, Em B, W B, Mh S. Uninformed consent in nutrigenomic research [Internet]. European journal of human genetics : EJHG. 2017 [cited 2020 Sep 21]. Available from: https://pubmed.ncbi.nlm.nih.gov/28488677/

50. Largent Ea, J K. Preclinical Alzheimer Disease and the Dawn of the Pre-Caregiver [Internet]. JAMA neurology. 2019 [cited 2020 Sep 21]. Available from: https://pubmed.ncbi.nlm.nih.gov/30855658/

51. Meulenbroek O, Vernooij-Dassen M, Kessels RPC, Graff MJL, Sjögren MJC, Schalk BWM, et al. Informed consent in dementia research. Legislation, theoretical concepts and how to assess capacity to consent. Eur Geriatr Med. 2010;1(1):58–63.

52. Dunn Lb, Bw P. When Does Therapeutic Misconception Affect Surrogates’ or Subjects’ Decision Making about Whether to Participate in Dementia Research? [Internet]. AMA journal of ethics. 2017 [cited 2020 Sep 21]. Available from: https://pubmed.ncbi.nlm.nih.gov/28813240/

53. Werner P, S S. Practical and Ethical Aspects of Advance Research Directives for Research on Healthy Aging: German and Israeli Professionals’ Perspectives [Internet]. Frontiers in medicine. 2018 [cited 2020 Sep 21]. Available from: https://pubmed.ncbi.nlm.nih.gov/29675415/

54. Kim SYH, Karlawish JH, Kim HM, Wall IF, Bozoki AC, Appelbaum PS. Preservation of the capacity to appoint a proxy decision maker: implications for dementia research. Arch Gen Psychiatry. 2011 Feb;68(2):214–20.

55. Olde Rikkert MG, van der Vorm A, Burns A, Dekkers W, Robert P, Sartorius N, et al. Consensus statement on genetic research in dementia. Am J Alzheimers Dis Other Demen. 2008;23(3):262–6.

56. Pachana NA , et al. Can we do better? Researchers’ experiences with ethical review boards on projects with later life as a focus. - PubMed - NCBI [Internet]. 2015 [cited 2017 Jan 5]. Available from: https://www-1ncbi-1nlm-1nih-1gov-1mf9loa7101b3.han.mh-hannover.de/pubmed/25374104

57. West E, Stuckelberger A, Pautex S, Staaks J, Gysels M. Operationalising ethical challenges in dementia research—a systematic review of current evidence. Age Ageing. 2017;1–10.

58. Forlini C. Patient preferences may be indicative of normative issues in dementia research. J Alzheimers Dis. 2017;59(1):11–2.

59. Hosie A, S K, N R, I G, D P, C S, et al. Older Persons’ and Their Caregivers’ Perspectives and Experiences of Research Participation With Impaired Decision-Making Capacity: A Scoping Review [Internet]. The Gerontologist. 2020 [cited 2020 Sep 21]. Available from: https://pubmed.ncbi.nlm.nih.gov/32866239/

60. Ries NM, Thompson KA, Lowe M. Including people with dementia in research: an analysis of Australian ethical and legal rules and recommendations for reform. J Bioethical Inq. 2017;14(3):359–74.

61. Davis DS. Ethical issues in Alzheimer’s disease research involving human subjects. J Med Ethics. 2017;medethics – 2016.

62. Hanson LC, Gilliam R, Lee TJ. Successful clinical trial research in nursing homes: the Improving Decision-Making Study. Clin Trials Lond Engl. 2010 Dec;7(6):735–43.

63. Kim SYH, Karlawish J, Berkman BE. Ethics of genetic and biomarker test disclosures in neurodegenerative disease prevention trials. Neurology. 2015 Apr 7;84(14):1488–94.

64. Jongsma KR , et al. Has dementia research lost its sense of reality? A descriptive analysis of eligibility criteria of Dutch dementia research protocols. - PubMed - NCBI. 2016 [cited 2017 Jan 6]; Available from: https://www-1ncbi-1nlm-1nih-1gov-1mf9loaxh009c.han.mh-hannover.de/pubmed/27323673

65. Pierce R. A changing landscape for advance directives in dementia research. Soc Sci Med. 2010 Feb;70(4):623–30.

66. Sherratt C, Soteriou T, Evans S. Ethical issues in social research involving people with dementia. Dementia. 2007;6(4):463–79.

67. Wall A. Including persons with Alzheimer disease in research on comorbid conditions. - PubMed - NCBI [Internet]. 2009 [cited 2017 Jan 5]. Available from: https://www-1ncbi-1nlm-1nih-1gov-1mf9loa7101b3.han.mh-hannover.de/pubmed/19241733

68. Karlawish J, Rubright J, Casarett D, Cary M, Ten Have T, Sankar P. Older Adults’ Attitudes Toward Enrollment of Non-competent Subjects Participating in Alzheimer’s Research. Am J Psychiatry. 2009 Feb;166(2):182–8.

69. Beattie E, O’Reilly M, Fetherstonhaugh D, McMaster M, Moyle W, Fielding E. Supporting autonomy of nursing home residents with dementia in the informed consent process. Dementia. 2019 Nov 1;18(7-8):2821–35.

70. Grill Jd, J K. Study partners should be required in preclinical Alzheimer’s disease trials [Internet]. Alzheimer’s research & therapy. 2017 [cited 2020 Sep 21]. Available from: https://pubmed.ncbi.nlm.nih.gov/29212555/

71. De Vries R, Stanczyk AE, Ryan KA, Kim SYH. A Framework for Assessing the Quality of Democratic Deliberation: Enhancing Deliberation as a Tool For Bioethics. J Empir Res Hum Res Ethics Int J. 2011 Sep;6(3):3–17.

72. Dubois M-F, Bravo G, Graham J, Wildeman S, Cohen C, Painter K, et al. Comfort with proxy consent to research involving decisionally impaired older adults: do type of proxy and risk-benefit profile matter? Int Psychogeriatr. 2011 Nov;23(09):1479–88.

73. Karlawish J, Kim SYH, Knopman D, van Dyck CH, James BD, Marson D. The Views of Alzheimer Disease Patients and Their Study Partners on Proxy Consent for Clinical Trial Enrollment. Am J Geriatr Psychiatry. 2008 Mar;16(3):240–7.

74. Novek S, Wilkinson H. Safe and inclusive research practices for qualitative research involving people with dementia: a review of key issues and strategies. Dementia. 2019;18(3):1042–59.

75. Ries N, E M, R S-F. Planning Ahead for Dementia Research Participation: Insights from a Survey of Older Australians and Implications for Ethics, Law and Practice [Internet]. Journal of bioethical inquiry. 2019 [cited 2020 Sep 21]. Available from: https://pubmed.ncbi.nlm.nih.gov/31297689/

76. Kim SYH, Kim HM, Knopman DS, De Vries R, Damschroder L, Appelbaum PS. Effect of public deliberation on attitudes toward surrogate consent for dementia research. Neurology. 2011 Dec 13;77(24):2097–104.

77. Largent Ea, Sp H, K H, Ak H, S J, Jc L, et al. Ethical and Regulatory Issues for Embedded Pragmatic Trials Involving People Living with Dementia [Internet]. Journal of the American Geriatrics Society. 2020 [cited 2020 Sep 21]. Available from: https://pubmed.ncbi.nlm.nih.gov/32589273/

78. Kutschenko LK. Diagnostic misconceptions? A closer look at clinical research on Alzheimer’s disease. J Med Ethics. 2012 Jan;38(1):57–9.

79. Christensen KD, Roberts JS, Uhlmann WR, Green RC. Changes to perceptions of the pros and cons of genetic susceptibility testing after APOE genotyping for Alzheimer disease risk. Genet Med. 2011;13(5):409–14.

80. Sperling RA, Karlawish J, Johnson KA. Preclinical Alzheimer disease—the challenges ahead. Nat Rev Neurol. 2013;9(1):54–8.

81. Grill JD, Zhou Y, Elashoff D, Karlawish J. Disclosure of amyloid status is not a barrier to recruitment in preclinical Alzheimer’s disease clinical trials. Neurobiol Aging. 2016 Mar;39:147–53.

82. Ienca M, Vayena E, Blasimme A. Big data and dementia: charting the route ahead for research, ethics, and policy. Front Med. 2018;5:13.

83. Arias Jj, Am T, Bj O, J K. The Proactive Patient: Long-Term Care Insurance Discrimination Risks of Alzheimer’s Disease Biomarkers [Internet]. The Journal of law, medicine & ethics : a journal of the American Society of Law, Medicine & Ethics. 2018 [cited 2020 Sep 21]. Available from: https://pubmed.ncbi.nlm.nih.gov/30147000/

84. van der Vorm A, Vernooij-Dassen MJFJ, Kehoe PG, Rikkert MGMO, van Leeuwen E, Dekkers WJM. Ethical aspects of research into Alzheimer disease. A European Delphi Study focused on genetic and non-genetic research. J Med Ethics. 2009 Feb 1;35(2):140–4.

85. van der Vorm A, Rikkert MO, Vernooij-Dassen M, Dekkers W, on behalf of the EDCON panel. Genetic research into Alzheimer’s Disease: a European focus group study on ethical issues. Int J Geriatr Psychiatry. 2008 Jan;23(1):11–5.

86. Pierce R. Complex calculations: ethical issues in involving at-risk healthy individuals in dementia research. J Med Ethics. 2010 Sep 1;36(9):553–7.

87. Shulman MB, Harkins K, Green RC, Karlawish J. Using AD biomarker research results for clinical care: a survey of ADNI investigators. Neurology. 2013 Sep 24;81(13):1114–21.

88. Garand L, Lingler JH, Conner KO, Dew MA. Diagnostic labels, stigma, and participation in research related to dementia and mild cognitive impairment. Res Gerontol Nurs. 2009 Apr;2(2):112–21.

89. Lange MM, Rogers W, Dodds S. Vulnerability in research ethics: a way forward. Bioethics. 2013;27(6):333–40.

90. Murphy K, Jordan F, Hunter A, Cooney A, Casey D. Articulating the strategies for maximising the inclusion of people with dementia in qualitative research studies. Dementia. 2014;1471301213512489.

91. Law E, Russ TC, Connelly PJ. What motivates patients and carers to participate in dementia studies? Emma Law and colleagues used the participation chain model to analyse why people took part in research, and why they might not want to co-operate in future. Nurs Older People. 2013;25(9):31–6.

92. Bittlinger M, S M. Opening the debate on deep brain stimulation for Alzheimer disease - a critical evaluation of rationale, shortcomings, and ethical justification [Internet]. BMC medical ethics. 2018 [cited 2020 Sep 21]. Available from: https://pubmed.ncbi.nlm.nih.gov/29886845/

93. Largent Ea, J K, Jd G. Study partners: essential collaborators in discovering treatments for Alzheimer’s disease [Internet]. Alzheimer’s research & therapy. 2018 [cited 2020 Sep 21]. Available from: https://pubmed.ncbi.nlm.nih.gov/30261910/

94. Grill JD, Karlawish J. Consider the Source: The Implications of Informant Type on Outcome Assessments. Alzheimer Dis Assoc Disord. 2015;29(4):364.

95. Stites Sd, K H, Jd R, J K. Relationships Between Cognitive Complaints and Quality of Life in Older Adults With Mild Cognitive Impairment, Mild Alzheimer Disease Dementia, and Normal Cognition [Internet]. Alzheimer disease and associated disorders. 2018 [cited 2020 Sep 21]. Available from: https://pubmed.ncbi.nlm.nih.gov/29944474/

96. Salmon D, Lineweaver T, Bondi M, Galasko D. Knowledge of APOE genotype affects subjective and objective memory performance in healthy older adults. Alzheimers Dement J Alzheimers Assoc. 2012;8(4):P123–4.

97. Caspi E. Trust at stake: Is the “dual mission” of the U.S. Alzheimer’s Association out of balance? [Internet]. Dementia (London, England). 2019 [cited 2020 Sep 21]. Available from: https://pubmed.ncbi.nlm.nih.gov/28840758/

98. Gove D, Diaz-Ponce A, Georges J, Moniz-Cook E, Mountain G, Chattat R, et al. Alzheimer Europe’s position on involving people with dementia in research through PPI (patient and public involvement). Aging Ment Health. 2018 Jun 3;22(6):723–9.

99. Morbey H, Harding AJ, Swarbrick C, Ahmed F, Elvish R, Keady J, et al. Involving people living with dementia in research: an accessible modified Delphi survey for core outcome set development. Trials. 2019;20(1):1–10.

100. Jongsma K, Bos W, van de Vathorst S. Morally Relevant Similarities and Differences Between Children and Dementia Patients as Research Subjects: Representation in Legal Documents and Ethical Guidelines: Children and Dementia Patients as Research Subjects. Bioethics. 2015 Nov;29(9):662–70.

101. Kim SYH, Uhlmann RA, Appelbaum PS, Knopman DS, Kim HM, Damschroder L, et al. Deliberative assessment of surrogate consent in dementia research. Alzheimers Dement. 2010 Jul;6(4):342–50.

102. Bittlinger M. Call of Duty at the Frontier of Research: Normative Epistemology for High-Risk/High-Gain Studies of Deep Brain Stimulation [Internet]. Cambridge quarterly of healthcare ethics : CQ : the international journal of healthcare ethics committees. 2018 [cited 2020 Sep 21]. Available from: https://pubmed.ncbi.nlm.nih.gov/30198469/

103. Korczyn A. Drug Trials in Dementia: Challenging Ethical Dilemmas. Curr Alzheimer Res. 2007 Sep 1;4(4):468–72.

104. Stocking CB, Hougham GW, Danner DD, Patterson MB, Whitehouse PJ, Sachs GA. Variable judgments of decisional capacity in cognitively impaired research subjects. J Am Geriatr Soc. 2008 Oct;56(10):1893–7.

105. Fletcher JR, Lee K, Snowden S. Uncertainties When Applying the Mental Capacity Act in Dementia Research: A Call for Researcher Experiences. Ethics Soc Welf. 2019;13(2):183–97.

106. van Duinkerken E, J F, J L-F, Mc D, J L, Dc M. Medical and Research Consent Decision-Making Capacity in Patients with Alzheimer’s Disease: A Systematic Review [Internet]. Journal of Alzheimer’s disease : JAD. 2018 [cited 2020 Sep 21]. Available from: https://pubmed.ncbi.nlm.nih.gov/30103326/

107. Jongsma K, J P, S S, K R. Motivations for people with cognitive impairment to complete an advance research directive - a qualitative interview study [Internet]. BMC psychiatry. 2020 [cited 2020 Sep 21]. Available from: https://pubmed.ncbi.nlm.nih.gov/32641010/

108. Overton E, Appelbaum PS, Fisher SR, Dohan D, Roberts LW, Dunn LB. Alternative decision-makers’ perspectives on assent and dissent for dementia research. Am J Geriatr Psychiatry. 2013;21(4):346–54.

109. Cary MS, Rubright JD, Grill JD, Karlawish J. Why are spousal caregivers more prevalent than nonspousal caregivers as study partners in AD dementia clinical trials? Alzheimer Dis Assoc Disord. 2015 Mar;29(1):70–4.

110. Jongsma KR, van de Vathorst S. Dementia research and advance consent: it is not about critical interests. J Med Ethics. 2015 Aug;41(8):708–9.
